# Stabilizing high-efficiency perovskite solar cells via strategic interfacial contact engineering

**DOI:** 10.1038/s41566-025-01791-1

**Published:** 2025-11-07

**Authors:** Guixiang Li, Zuhong Zhang, Benjamin Agyei-Tuffour, Luyan Wu, Thomas W. Gries, Karunanantharajah Prashanthan, Lennart Frohloff, Artem Musiienko, Jinzhao Li, Rui Zhu, Lucy J. F. Hart, Luyao Wang, Zhe Li, Bo Hou, Norbert Koch, Michele Saba, Piers R. F. Barnes, Jenny Nelson, Paul J. Dyson, Mohammad Khaja Nazeeruddin, Meng Li, Antonio Abate

**Affiliations:** 1 School of Materials Science and Engineering, Southeast University, Nanjing, China; 2 Key Lab for Special Functional Materials of Ministry of Education, National & Local Joint Engineering Research Center for High-efficiency Display and Lighting Technology, School of Nanoscience and Materials Engineering, Collaborative Innovation Center of Nano Functional Materials and Applications, Henan University, Kaifeng, China; 3 Helmholtz-Zentrum Berlin für Materialien und Energie GmbH, Berlin, Germany; 4 Department of Materials Science and Engineering, School of Engineering Sciences, College of Basic and Applied Sciences, University of Ghana, Legon, Ghana; 5 Department of Physics, University of Cagliari, Monserrato, Italy; 6 Department of Physics, University of Jaffna, Jaffna, Sri Lanka; 7 Department of Physics, Humboldt University of Berlin, Berlin, Germany; 8 Department of Physics, Imperial College London, London, UK; 9 State Key Lab for Physical Chemistry of Solid Surfaces, Department of Chemistry, College of Chemistry and Chemical Engineering, Pen-Tung Sah Institute of Micro-Nano Science and Technology, Xiamen University, Xiamen, China; 10 School of Engineering and Materials Science (SEMS), Queen Mary University of London, London, UK; 11 School of Physics and Astronomy, Cardiff University, Cardiff, UK; 12 Institute of Chemical Sciences and Engineering, École Polytechnique Fédérale de Lausanne (EPFL), Lausanne, Switzerland; 13 Mechanical and Energy Engineering Department, College of Engineering, Imam Abdulrahman Bin Faisal University, Dammam, Saudi Arabia; 14 Department of Chemistry, Bielefeld University, Bielefeld, Germany; 15 Department of Chemical, Materials and Production Engineering, University of Naples Federico II, Naples, Italy

**Keywords:** Solar cells, Photonic devices

## Abstract

Surface passivation in perovskite solar cells can enhance device efficiency, yet incomplete interfacial functionality poses challenges to long-term reliability. Here we present a strategic interfacial engineering approach using sodium heptafluorobutyrate to fully functionalize the perovskite surface. Sodium heptafluorobutyrate acts as an ion shield that tunes the perovskite surface work function and increases the defect formation energy, resulting in an improved interface with the electron transport layer that minimizes recombination and boosts electron extraction under operation. We find that a sodium-heptafluorobutyrate-functionalized perovskite surface promotes a uniform, compact C_60_ layer that effectively blocks ion diffusion and stabilizes the device stack. This approach allows p–i–n perovskite solar cells to achieve a record power conversion efficiency (PCE) of 27.02% (certified 26.96% with a maximum-power-point-tracking PCE of 26.61%). Devices with an active area of 1 cm^2^ deliver a PCE of 25.95%. Perovskite solar cells retain 100% of their initial efficiency following 1,200 h of continuous 1-sun illumination at the maximum power point. Devices also demonstrate exceptional thermal stability, retaining 92% of the initial PCE when ageing at 85 °C for 1,800 h and 94% after 200 thermal cycles between –40 °C and +85 °C.

## Abstract (web summary)

Engineering the perovskite–electrical contact interface with sodium heptafluorobutyrate reduces interfacial defects and improves charge transport in perovskite solar cells. Functionalized devices deliver a certified power conversion efficiency of 26.96%, which is fully retained after 1,200 h of continuous operation under 1-sun illumination.

## Main

Metal halide perovskite solar cells (PSCs) have impressive power conversion efficiencies (PCEs)^1,2^ due to their strong light absorption and appreciably high carrier mobility, and their low cost makes them particularly attractive^3,4^. Inverted (p–i–n) PSCs have achieved almost 27% PCE, competitive with silicon-based solar cells^5,6^. Such device architectures possess inherently better compatibility with flexible substrates and low-temperature processing, which could facilitate scale-up and the manufacture of tandem cells^7–9^. To drive forward the industrialization of perovskite photovoltaics, however, device reliability is a critical issue that is increasingly receiving attention^10^.

In the p–i–n architecture, the upper interface involves contact between the perovskite surface and the electron-selective contact (ESC). The surface of perovskite films often exhibit a high abundance of defects, primarily due to the formation of volatile compounds during the fabrication process, leaving behind defects^11,12^. These defects, in turn, lead to notable non-radiative recombination losses^13–15^. When illuminated or operated at elevated temperatures, the weaker bonds within the perovskite readily dissociate, generating vacancies and interstitial defects that migrate towards the metal contacts, posing a significant challenge to device stability^16,17^. Typically, the suppression of ion migration relies on passivating undercoordinated sites within the perovskite lattice. Nonetheless, the ultimate barrier to prevent ion migration within a device often lies at the ESC interface. Fullerene-type materials such as carbon 60 (C_60_) are commonly used as the ESC, which tend to aggregate when operated at elevated temperatures^18^. Consequently, not only charge transport is hampered but also the ability of the ESC to block reactions between the metal electrode and the underlying perovskite layer is undermined, thereby compromising device durability^16^. Therefore, achieving an optimal interface contact requires (1) the passivation of surface defects and stabilization of surface structure in the perovskite, (2) favourable interface charge transport and (3) a compact and stable ESC that blocks ion mobility. By creating a superior upper interface, the operational stability of PSCs should be prolonged.

To address these issues, we consider sodium heptafluorobutyrate (SHF), combining the perfluorous tail and carboxylate head, offering dual functionality as a candidate. SHF has the potential to form a robust hydrophobic barrier, simultaneously providing the effective passivation of surface defects. Using SHF, we present an advanced perovskite surface-to-ESC integrated interfacial engineering strategy. SHF increases the defect formation energy of the perovskite surface, stabilizing undercoordinated Pb(II) and eliminating the non-photoactive phase. Due to suitable dipole characteristics, SHF induces carrier redistribution and tunes the perovskite surface work function (WF) to enhance the open-circuit voltage. During ESC deposition, SHF causes C_60_ to stack in densely packed layers, which suppresses ion diffusion within the device. The resulting PSCs exhibit a record PCE of 27.02% (certified PCE being 26.96% with a stabilized PCE of 26.61%). Additionally, 1-cm^2^ devices show a PCE of up to 25.95%. In particular, the SHF-treated device retains its initial PCE (0% efficiency loss) after 1,200 h of maximum power point tracking (MPPT) under 1-sun illumination (International Summit on Organic Photovoltaic Stability ISOS-L-1) and demonstrated over 92% retention when aged at ~85 °C (ISOS-D-2) over 1,800 h. The device cycled between −40 °C and 85 °C showed a retention of 94% of its initial PCE after 200 cycles (ISOS-T-3).

## Functionalization of the perovskite surface with SHF

**Fig. 1:**
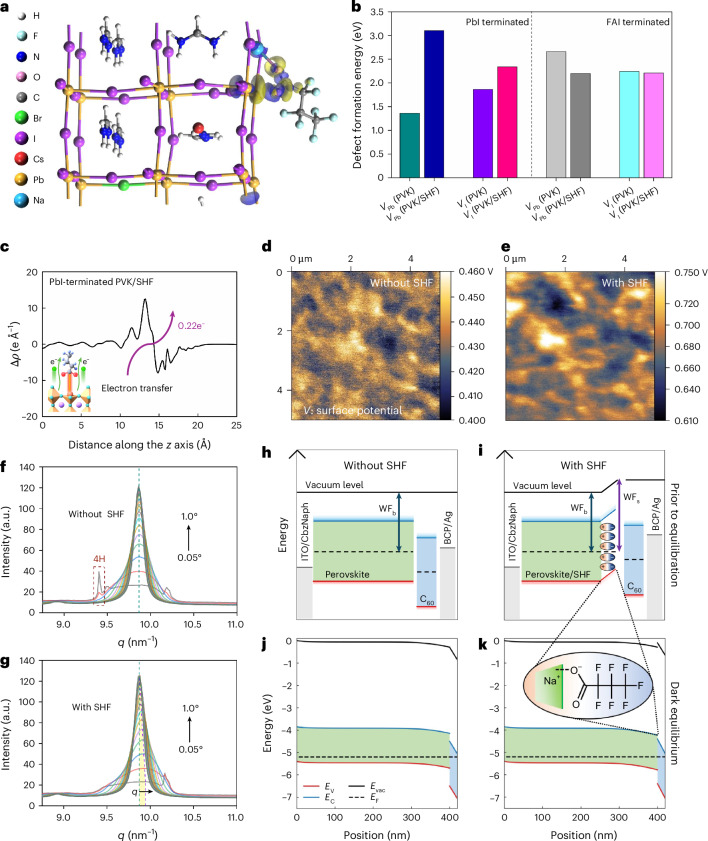
Perovskite surface structure and properties. **a**, Constructed SHF adsorption model on the perovskite structure. **b**, Defect formation energy of the perovskite surface with and without SHF adsorption. PVK, perovskite. **c**, Planar Δ*ρ* for the PbI_2_-terminated surface of single SHF-adsorbed perovskite. **d**,**e**, KPFM maps of perovskites without (**d**) and with (**e**) SHF. The colour scale represents the surface potential (*V*). Image size, 5 μm × 5 μm. **f**,**g**, Integrated GIWAXS patterns on (001) peaks of perovskites without (**f**) and with (**g**) SHF for incident angles from 0.05° to 1.0°. **h**–**k**, Simulated energy-level diagrams of control (**h** and **j**) and SHF-containing (**i** and **k**) devices before and after dark equilibration. The SHF layer introduces a vacuum-level shift and enhances the electric field across C_60_, consistent with an increased built-in potential. WF_b_, bulk work function; WF_s_, surface work function; *E*_F_ is the Fermi level energy; *E*_C_ and *E*_V_ are the conduction and valence band edges, respectively, and *E*_vac_ is the vacuum energy level.

To improve the surface electronic properties of perovskites in a targeted manner, we studied their defect characteristics through first-principles DFT calculations (Fig. 1a). We compared the defect formation energy (*V*_Pb_ and *V*_I_) on two types of perovskite surface termination (PbI and formamidinium iodide (FAI)), with the structure models shown in Supplementary Figs. 1–4. The computation revealed that the formation energy of a Pb vacancy is smaller than that of an I vacancy for the PbI-terminated surface, with both being less than that for an FAI-terminated surface (Fig. 1b). This suggests that defects are more easily formed on a pure PbI-terminated surface compared with a pure FAI-terminated surface, corresponding to lower surface stability. In particular, to aid crystal growth and defect passivation, a lead(II) iodide (PbI_2_)-rich stoichiometry is often used in perovskite fabrication, which also generates a PbI-terminated surface^19,20^, probably undermining the operational stability of PSCs.

SHF was used to functionalize the perovskite surface via a post-treatment strategy (Fig. 1a and Supplementary Figs. 2–4). DFT calculations show that in the surface adsorption system, the formation energy of vacancies on the PbI surface significantly increases (Fig. 1b), indicating that SHF plays a crucial role in enhancing the surface stability. Mulliken charge population calculations indicate that surface adsorption per SHF will result in 0.22 electron transfer to SHF (Fig. 1c), amplified on the SHF-stacking surface (Supplementary Figs. 5–7).

SHF consists of Na^+^ and a perfluorocarbon chain terminated with a COO^−^ group, forming a system with a dipole moment. On interacting with the perovskite surface, Na^+^ introduces localized positive charges, whereas the COO^−^ group generates localized negative charges. Due to their electrostatic interaction and the large size of perfluorocarboxylate anions, Na^+^ ions potentially remain at the perovskite surface beside the COO^−^ groups^21^. This combination would establish an interfacial dipole pointing from SHF towards the perovskite. Kelvin probe force microscopy (KPFM) confirms that SHF alters the surface WF of the perovskite, validating the dipole effect. Figure 1d,e shows the potential distribution patterns of control and SHF-treated perovskite films. The average contact potential difference value increases from 0.43 to 0.68 eV after treating the perovskite with SHF (Supplementary Fig. 8). This increase is consistent with the electrostatic potential and WF estimated from DFT (Supplementary Figs. 9–11). We further reveal that the appropriate SHF coverage is a key factor in tuning the surface WF (Supplementary Fig. 12). In this case, the formed positive dipoles at the perovskite–ETL interface increases the PSC’s built-in potential^22–24^ (Fig. 1h,i and Supplementary Fig.  13). To clarify the impact of SHF-induced dipoles under device-relevant conditions, we carried out drift-diffusion simulations on full device stacks (Fig. 1h–k). Although the perovskite band structure remains largely unaffected in the dark equilibrium state, the introduced SHF significantly enhances the internal electric field across the undoped C_60_ layer. This originates from reduced energetic offsets at the perovskite–C_60_ interface, consistent with a larger built-in potential. The enhanced interfacial field is expected to promote charge extraction and increase the device’s open-circuit voltage (*V*_OC_). To validate the role of molecular dipoles, control experiments were performed using sodium acetate (NaOAc; CH_3_COONa) under identical processing conditions. As shown in Supplementary Fig. 14, NaOAc treatment led to a minor change in the surface potential, in contrast to the pronounced shift observed with SHF. To understand the molecular origin of the observed dipole difference, we performed DFT calculations on SHF and NaOAc. The structures were optimized using the wB97XD functional and Def2TZVP basis set. The calculated dipole moment of SHF is 8.97 D, significantly larger than that of NaOAc (5.91 D; Supplementary Fig. 15), highlighting the contribution of the fluorinated tail in enhancing the overall molecular polarity.

The interaction between SHF and perovskite was further supported by Fourier transform infrared spectra, X-ray photoelectron spectra and nuclear magnetic resonance spectra^25,26^ (Supplementary Figs. 16–18). Grazing-incidence wide-angle X-ray scattering (GIWAXS) was used to characterize the surface crystal structure of the perovskite films. At low incidence angles (<1.0°; Fig. 1f), a sharp peak at *q* ≈ 9.4 nm^−1^ is observed in the untreated film, indexed as the hexagonal polytype 4H (ref. ^27^). In particular, SHF treatment eliminates the 4H phase (Fig. 1h). Additionally, a shift in the diffraction peak of the (001) crystal plane for the SHF-treated perovskite is observed, originating from the film surface (Fig. 1g). The shift decreases as the diffraction angle increases, indicative of a surface-specific interaction between SHF and the perovskite^28^ (Supplementary Fig. 19). According to the Bragg equation, *q* = 4πsin*θ*/*λ* = 2π/*d*, where *d* is the Bragg spacing in scattering, a diffraction peak position shifting to higher angles corresponds to a contraction of the lattice^29^. This helps stabilize the perovskite surface structure due to the improved Goldschmidt tolerance factor^30^. Note that the interaction of SHF with the perovskite surface is further supported by X-ray diffraction (Supplementary Fig. 20).

## Structure and optoelectronic properties of interfacial contact

**Fig. 2:**
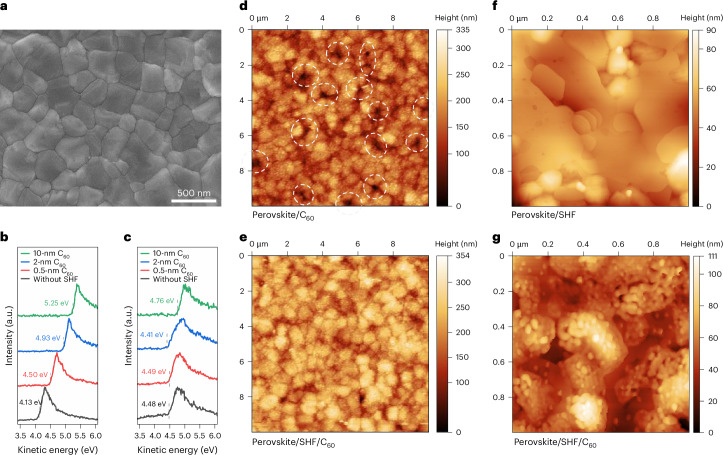
Optoelectronic properties between the perovskite and ESC. **a**, SEM image of SHF-treated perovskite films. **b**,**c**, Ultraviolet photoelectron spectroscopy spectra of the cut-off region for C_60_ with different deposition thicknesses on the perovskite films without (**b**) and with (**c**) SHF treatment. **d**,**e**, AFM images of perovskite/C_60_ (**d**) and perovskite/SHF/C_60_ (10-nm C_60_ deposited; **e**). Image size, 10 μm × 10 μm. **f**,**g**, AFM images of perovskite/SHF (**f**) and perovskite/SHF/C_60_ (**g**) samples (the AFM scans were taken from different areas (**f**) with one-half of the sample covered during deposition and 2-nm C_60_ deposited only on the other half (**g**)). Image size, 1 μm × 1 μm. The colour scales in **d**–**g** represent the surface height (nm).

Since the ESC coats the perovskite surface, its structural characteristics are affected by the underlying perovskite layer, impacting the device performance. We monitored the perovskite film morphology using scanning electron microscopy (SEM) and atomic force microscopy (AFM). The improved perovskite surface due to SHF treatment was confirmed by SEM and AFM images (Fig. 2a and Supplementary Figs. 21 and 22), which facilitated a better interface contact with the ESC. Subsequently, the structural and optoelectronic properties of ESC as a function of the thickness of the C_60_ layer was studied^31,32^. In the SHF-treated device, the alignment of the electronic levels is significantly improved, evident from the reduced steepness of the secondary electron cut-off for the perovskites in contact with C_60_ (Fig. 2b,c). The reduced slope variation indicates that the individual secondary electron cut-offs at different kinetic energies tend to align more consistently. This is attributed to the enhanced perovskite surface uniformity, which promoted more favourable C_60_ deposition. The valence band scans of the C_60_ layers indicate their contribution to the total spectrum (Supplementary Fig. 23). The data for the C_60_ layers were fitted as the sum of the pristine perovskite valence band signal and the signal from 10-nm-thick C_60_, allowing the perovskite and C_60_ contributions to be separated. The reduced interfacial energy barrier (Supplementary Figs. 24 and 25) would enhance the PSC performance, as we show below using drift-diffusion simulations.

The stacking properties of the deposited C_60_ layer were progressively probed using AFM. At a 10 × 10 μm^2^ scale, 10-nm C_60_ deposited on the control film surface exhibited noticeable voids (Fig. 2d). By contrast, C_60_ on the SHF-treated perovskite surface formed a densely packed layer (Fig. 2e). This difference suggests that the SHF layer mediates C_60_ growth and provides a favourable interfacial contact between the perovskite and ESC. The SHF probably reduces the surface energy and allows C_60_ to form a more uniform layer. By contrast, when non-polar molecules such as C_60_ are deposited directly onto hydrophilic surfaces like perovskites, they tend to aggregate to minimize unfavourable surface interaction energy^33,34^. As the thickness decreases into 2-nm C_60_, the differences between control and treated surfaces became more apparent, probably due to the direct effect of the initially deposited thin C_60_ layer contacting with the underlying perovskite surface (Supplementary Fig. 26). To gain deeper insights, the growth of a 2-nm C_60_ layer on SHF-treated perovskite surface was monitored at a 1 × 1 μm^2^ scale, with the perovskite film partially covered as a control (Fig. 2f). We observed that C_60_ tends to grow in island-like forms consisting of particle aggregates, showing an uneven structure (Fig. 2g). Interestingly, these islands are not isolated, but well interconnected, forming a cohesive layer.

## Carrier dynamics and photovoltaic performance

**Fig. 3:**
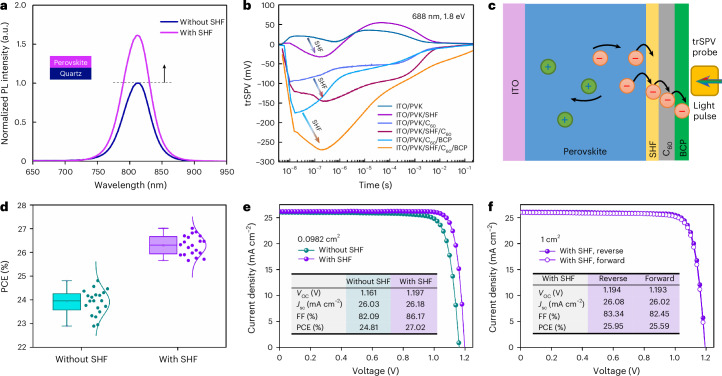
Electron extraction in PSCs and device performance. **a**, Steady-state PL spectra for the untreated and SHF-treated perovskite films. The *y* axis shows the normalized PL intensity. **b**, trSPV charge dynamics at the perovskite–ESC interfaces. **c**, Schematic describing the charge separation mechanisms. **d**, PCE distribution of control and SHF-treated PSCs, collected from 20 independent devices per condition (*n* = 20). Box plots show the interquartile range with the median as the centre line; whiskers indicate the 5th–95th percentiles. **e**,**f**, *J*–*V* curves of untreated and SHF-treated PSCs with working areas of 0.0982 cm^2^ (**e**) and 1 cm^2^ (**f**).

To provide insights into the changes in electronic states, steady-state photoluminescence (PL) measurements were performed, where an enhanced PL intensity indicates the decreased defect-induced non-radiative recombination^9^ (Fig. 3a). Additionally, we observed an improvement of over two times in the PL quantum yield after SHF treatment (Supplementary Fig. 27), indicating that SHF effectively enhanced passivation. After contacting the C_60_ layer, the SHF-treated stack (perovskite/SHF/C_60_) retained a higher PL quantum yield (nearly three times) compared with the control (perovskite/C_60_), demonstrating reduced interfacial losses and improved optoelectronic quality.

To evaluate the charge transport properties in PSCs, transient surface photovoltage (trSPV) measurements were conducted, exciting the sample with a 5-ns laser pulse with a photon energy of 1.8 eV and fluences of 0.010 μJ cm^−2^, inducing a charge carrier density equivalent to 1 sun (refs. ^35–37^). The trSPV signal amplitude is proportional to the concentration of electrons extracted to the ESC, expressed as SPV(*t*) ≈ Δ*n*_ESC_(*t*), where *t* denotes time^37^. The untreated perovskite sample without an ESC exhibits a positive trSPV signal due to hole accumulation near the perovskite surface, which can potentially limit electron extraction (Fig. 3b). By contrast, the SHF layer in the treated perovskite induces a negative trSPV signal, highlighting a qualitative change in the charge separation behaviour, with electrons dominating near the ESC side (Fig. 3c). The effect of SHF on the indium tin oxide (ITO)/perovskite/C_60_ and ITO/perovskite/C_60_/bathocuproine (BCP) samples was further investigated (Fig. 3b). SHF treatment considerably increases the amplitude and rise speed, with the peak amplitude for perovskite/SHF/C_60_/BCP being about twice as large as the untreated sample. This increase demonstrates a substantial improvement in electron extraction. The rise in amplitude, particularly in the post-extraction region (1–100 μs), indicates that SHF helps to align the energy-level alignment and passivate the perovskite surface. Additionally, similar trSPV results were observed for the perovskite–ESC interface on quartz, with an even more pronounced boost in rise speed and amplitude due to SHF treatment (Supplementary Figs. 28–30). Dark current and electrochemical impedance spectroscopy further indicate better interfacial contact following SHF treatment, leading to superior charge transport (Supplementary Figs. 31 and 32).

**Fig. 4:**
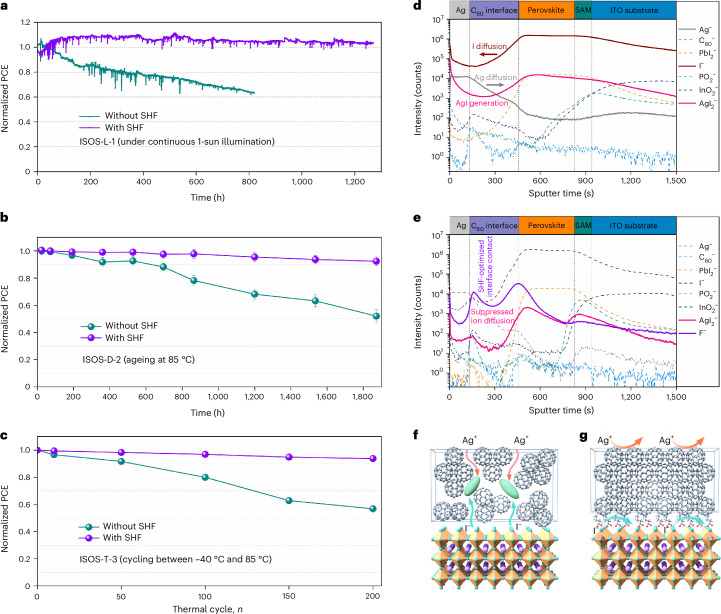
PV performance and stabilization mechanism of the devices. **a**, MPPT of encapsulated control and SHF-treated devices under simulated 1-sun illumination (ISOS-L-1I) in air (initial PCEs of 23.7% and 25.2%, respectively). **b**, Thermal stability of control and SHF-treated devices aged at 85 °C (ISOS-D-2I). Initial average PCEs of 24.1% and 25.9% (*n* = 3 independent devices per condition; error bars represent standard deviation). **c**, Thermal cycling stability of encapsulated devices aged between −40 °C and 85 °C (ISOS-T-3) (initial PCEs of 24.5% and 26.4%). **d**,**e**, ToF-SIMS ion species depth profiles of Ag^−^ (Ag), ${{\rm{C}}}_{60}^{-}$ (C_60_), ${{\rm{PbI}}}_{2}^{-}$ (perovskite), I^−^ (perovskite), ${{\rm{PO}}}_{2}^{-}$ (SAM), ${{\rm{InO}}}_{2}^{-}$ (ITO), ${{\rm{AgI}}}_{2}^{-}$ (AgI) and F^−^ (SHF) of control (**d**) and SHF-treated (**e**) devices after thermal ageing. **f**,**g**, Ion diffusion schematics within control (**f**) and SHF-treated (**g**) devices.

PSCs were fabricated with a configuration comprising transparent conducting oxide substrate/self-assembled monolayers (SAMs) (4-(7H-dibenzo[*c*,*g*]carbazol-7-yl)butyl)phosphonic acid (CbzNaph)/perovskite/C_60_/BCP/silver (Ag; Supplementary Fig. 33). Statistical analysis (Fig. 3d) shows an overall higher PCE distribution for SHF-treated PSCs compared with control (untreated) PSCs. Statistics for *V*_OC_, short-circuit current density (*J*_sc_) and fill factor (FF) are presented in Supplementary Fig. 34, confirming the concentrated parameter distributions in PSCs, highlighting the effectiveness of SHF treatment on performance and reproducibility^38^. The champion control device exhibits a PCE of 24.81%, with a *V*_OC_ of 1.161 eV and FF of 82.09% (Fig. 3e). In comparison, the SHF-treated PSCs show a champion PCE of 27.02%, along with an increased *V*_OC_ of 1.197 eV and FF of 86.17% (Supplementary Fig. 35 and Supplementary Table 1), as well as reduced photocurrent–voltage (*J*–*V*) hysteresis^39^ (Supplementary Fig. 36 and Supplementary Table 2). The external quantum efficiency (EQE) spectra (Supplementary Fig. 37) show that the closed EQE onset are essentially the same in the devices. The integrated *J*_sc_ values match well with the values acquired from the *J*–*V* results (<1.3% discrepancy)^40,41^. Devices treated with NaOAc exhibited moderate performance improvements compared with the control but remained significantly inferior to SHF-treated devices (Supplementary Fig. 38). The laboratory-tested champion device was evaluated at the Fujian Metrology Institute, an accredited independent PV calibration laboratory, for independent validation. The device achieved a certified PCE of 26.96% under an aperture area of 7.82 mm^2^, with a certified MPPT efficiency of 26.61% (Supplementary Figs. 39 and 40). Additionally, a device with a 1-cm^2^ active area achieved a record PCE of 25.95% (Fig. 4f).

To gain further insights into the influence of SHF treatment on photovoltaic performance, the devices were simulated using Driftfusion^42^ (Supplementary Table 3). As shown in Supplementary Fig. 41, the simulation’s parameters increased the steady-state *V*_OC_, FF and PCE by amounts comparable with those observed experimentally. In the control device, which has a smaller built-in potential, the direction of the electric field in the perovskite bulk reverses at a lower applied voltage. This has a negative impact on *V*_OC_ and FF due to hole accumulation at the perovskite–C_60_ interface^43^ (Supplementary Fig. 42a). Conversely, when SHF is present, the resulting interfacial dipole improves the energetic alignment between the perovskite and C_60_, increasing the device’s built-in potential. Consequently, there is less hole accumulation at the perovskite–C_60_ interface at a given applied voltage, resulting in higher *V*_OC_ and FF (Supplementary Fig. 42b). To assess the effect of Fermi-level variation, we simulated energy band profiles by adjusting the Fermi levels of the perovskite and C_60_ layers (Supplementary Figs. 43 and 44). The results suggest that the SHF-induced dipole dominates the interfacial energy alignment.

## Operational stability of PSCs

Following the ISOS-L-1I protocol, the device stability was evaluated under simulated 1-sun illumination at the maximum power point^44^. The SHF-treated device demonstrated stable power output without PCE degradation after 1,200 h of continuous operation (Fig. 4a). In comparison, the control device decayed to ~60% of its initial value within only 800 h. The thermal stability of the devices was also assessed at 85 °C according to the ISOS-D-2I protocol (Fig. 4b), with the SHF-optimized device retaining 92% of its initial PCE after 1,800 h, whereas the control device degraded to approximately 52% of its initial PCE over the same period. Following the ISOS-T-3 protocol, devices were cycled between −40 °C and 85 °C. The SHF-treated device retained 94% of its initial PCE after 200 cycles, significantly outperforming the control device, which retained only 57% of its PCE (Fig. 4c). As expected, the fluorous chain in SHF provides a hydrophobic barrier (Supplementary Fig. 45), and hence, the stability of unencapsulated devices in a humid atmosphere was investigated (Supplementary Fig. 46).

To understand the mechanism behind the enhanced stability, the elemental distribution of aged devices was determined by time-of-flight secondary ion mass spectrometry (ToF-SIMS). From Fig. 4d and Supplementary Fig. 47a, in the control device, a substantial diffusion of I^−^ ions towards the Ag electrode is observed, where they react to form AgI, with Ag^+^ ions concurrently diffusing into the perovskite layer. For the SHF-treated device, ion migration is considerably suppressed, with the amount of AgI detected being around two orders of magnitude lower than that in the control device (Fig. 4e and Supplementary Fig. 47b). Since ion migration leads to perovskite decomposition^45,46^, their differences could be related to device stability. Indeed, the diffusion of I^−^ ions through ESC to Ag electrode, where it reacts to form AgI, further accelerates the degradation process (Fig. 4f). The SHF layer stabilizes the perovskite–ETL interface, blocking the passage of I^−^ ions to prolong the operational stability of the device (Fig. 4g). Additionally, SHF inherently possesses high thermal stability, with an initial decomposition temperature reaching up to 260 °C (Supplementary Fig. 48). Comparison with ToF-SIMS data from the pre-aged sample demonstrates that SHF maintains its stability over time, confirming its sustained effectiveness during device operation (Supplementary Fig. 49).

## Discussion

We report efficient and stable PSCs by anchoring a perfluorinated ionic barrier between the perovskite and ESC, which delivers PCEs of up to 27.02% and a certified PCE of 26.96% (certified MPPT PCE of 26.61%). SHF treatment increases the defect formation energy, eliminates non-photoactive phases and tunes the perovskite surface WF, effectively reducing the interfacial energetic offset between the perovskite and C_60_. The favourable surface structure provided by SHF also facilitates the growth of C_60_ layers, resulting in a denser ESC, which inhibits mobile ion shuttling within the device. Under accelerated ageing test conditions, the devices exhibit enhanced operational stability. These findings pave the way to the next generation of high-efficiency and high-stability perovskite-based optoelectronic devices.

## Methods

### Materials

FAI (99.99%, trace elements basis), methylammonium iodide (99.99%, trace elements basis) and methylammonium chloride (99.99%, trace elements basis) were purchased from Dyenamo. PbI_2_ (99.99%, trace metals basis) was purchased from TCI. Self-assembly molecules of CbzNaph (>99%) were purchased from Luminescence Technology. Caesium iodide (99.999%, AB 109298) was purchased from abcr Gute Chemie and used without further purification. Phenethylammonium bromide was obtained from Greatcell Solar Materials. C_60_ (≥99.99%, OE) was provided by CreaPhys. BCP (99.8%) was acquired from Ossila. Ag shots (2–3 mm, 99.999%) were bought from Alfa Aesar. SHF (C_4_F_7_NaO_2_) was obtained from Santa Cruz Biotechnology. Dimethyl sulfoxide-d_6_ (99.9 at.% D), *N*,*N*-dimethylformamide-d_7_ (≥99.5 at.% D), isopropyl alcohol, absolute ethanol and anhydrous chlorobenzene were acquired from Sigma-Aldrich.

### Solution, film and device fabrication

The patterned ITO, fluorine-doped tin oxide and quartz as substrates were cleaned with deionized water, acetone and isopropyl alcohol in this order for 15 min. The substrates were further exposed to ultraviolet–ozone for 30 min. The self-assembly molecule CbzNaph (0.3 mg ml^−1^) was dissolved in absolute ethanol and used as a hole-selective contact. The SAM solution was spin coated on the substrate at 3,000 rpm in a two-step process with 3-s acceleration and then kept for 30 s. Then, the substrate–SAM was transferred onto a hotplate and annealed at 100 °C for 10 min in a N_2_-filled glovebox. The perovskite precursor solution was prepared by dissolving 1.55 M of Cs_0.05_FA_0.9_MA_0.05_PbI_3_ with 3% excess PbI_2_ in dimethylformamide/dimethyl sulfoxide with a volume ratio of 4:1. Then, 10 mg ml^−1^ of methylammonium chloride was added to the precursor solution to improve the film morphology. The mixture was stirred overnight and then filtered through a 0.22-µm polytetrafluoroethylene membrane before use. The precursor solution was spin coated at 1,000 rpm for 10 s, followed by 5,000 rpm for 40 s. In the last 5 s during the spin-coating procedure, 200 μl of chlorobenzene as the antisolvent was dropped onto the substrate. The precast films were then annealed at 100 °C for 30 min to obtain perovskite films. For initial surface passivation, 1 mg ml^−1^ of phenethylammonium bromide was subsequently used, dissolved in isopropyl alcohol/dimethyl sulfoxide (195:5, v/v), which was spin coated on the prepared perovskite films at 5,000 rpm for 30 s and then annealed at 100 °C for 10 min. The fabricated perovskite encompassing two- and three-dimensional structures was used as a control. SHF was dissolved in isopropyl alcohol (in varying concentrations of 1, 3 and 5 mM) and spin coated onto the film surface, under 3,000 rpm for 30 s at a ramp of 1,000 rpm s^−1^ and then annealed at 100 °C for 5 min. The samples were then transferred into a thermal evaporator, where 23-nm C_60_, 7-nm BCP and 100-nm Ag layers were thermally deposited in a sequential manner, where C_60_ and BCP were evaporated by temperature control and the Ag electrode was evaporated by power control. The evaporation was carried out under a vacuum pressure of around 1.0 × 10^−6^ mbar at an initial deposition rate of 0.1 nm s^−1^. For stability tests, 20 nm of SnO_2_ was deposited as a barrier layer by thermal atomic layer deposition. The device area was determined by a shadow mask during metal evaporation. The active areas of the laboratory-test solar cells are 0.0982 cm^2^ and 1 cm^2^, and the aperture area for certification is 0.0782 cm^2^.

### Characterization

X-ray diffraction patterns were recorded using a PANalytical X’Pert Pro X-ray powder diffractometer with Cu Kα radiation (*λ* = 1.5419 Å). Field-emission SEM images were obtained on a SEM SUPRA 40 electron microscope. Fourier transform infrared spectra were recorded on a JASCO 4100 spectrometer. X-ray photoelectron spectroscopy was conducted on a Shimadzu AXIS SUPRA+ instrument. Water contact angles of the perovskite films were measured using a JC000DI optical contact-measuring system. Solution-state nuclear magnetic resonance spectra were acquired on Bruker Topspin v. 2.1 (AV400) and v. 3.0 (AV500). PL was measured on a laboratory-built PL setup, installed with a reflectance probe and a fibre-optic spectrometer from Ocean Insight, using LabVIEW software. AFM was performed on an NX10 system, Park Systems (XE-70). The AFM was kept in a glovebox with a N_2_ atmosphere. KPFM measurements were performed on Bruker MultiMode with NanoScope 9.1, Gwyddion, following the lift-mode amplitude-modulated KPFM in a N_2_ atmosphere in the dark using the following settings: *f*_0_ = 300 kHz, *k* = 40 N m^−1^ and *L* = 125 mm (Bruker RTESP-300). Kelvin probe was carried out for WF analysis using a Kelvin probe setup with a vibrating gold mesh driven by a piezoelectric 26 crystal (Kelvin probe S and CPD controller from Besocke Delta Phi).

### Photoelectron spectroscopy measurements

Photoemission spectra were recorded using a hemispherical electron analyser (SPECS Phoibos 100) in an ultrahigh-vacuum system equipped with a monochromated He I (*hν* = 21.218 eV) source for ultraviolet photoelectron spectroscopy and a standard Mg Kα (*hν* = 1,253.6 eV; anode power, 30 W) X-ray source for X-ray photoelectron spectroscopy. The base pressure of the analysis chamber was maintained at 1 × 10^−9^ mbar. All spectra were recorded at room temperature and under normal emission to the sample. For measuring the secondary electron cut-off, a sample bias of −10 V was used to overcome the analyser WF. A Solux MR16 4700K white halogen lamp (intensity equivalent to 150 mW cm^−2^) was used to examine any light-induced energy-level changes at the interfaces.

### Photovoltaic performance measurements

The *J*–*V* data were recorded under 1-sun-equivalent illumination using a wavelabs SINUS-70 LED class AAA solar simulator in a N_2_-filled glovebox. The light intensity was calibrated with a filtered KG3 silicon reference cell from the Fraunhofer-Institut für Solare Energiesysteme ISE. *J*–*V* scans were performed with a Keithley 2400 source measure unit, controlled by a measurement program written in LabVIEW. The stabilized power outputs under the maximum power points were tracked under illumination, and the PSCs were biased at the voltage at the maximum power point. Thermal cycling tests were conducted with a 10-min dwell and a ramp rate of 100 °C h^−1^. The EQE spectra were measured for PSCs, where fluorine-doped tin oxide was used in the devices. The EQE was obtained on an Oriel Instruments QEPVSI-b system integrated with a Newport 300-W xenon arc lamp, which was controlled using TracQ-Basic software. The system was equipped with a chopper and monochromatic light of Newport Cornerstone 260 for filtering the white light. The EQE setup was calibrated through a standard silicon photodetector before measurements.

### Reporting summary

Further information on research design is available in the Nature Portfolio Reporting Summary linked to this article.

## Online content

Any methods, additional references, Nature Portfolio reporting summaries, source data, extended data, supplementary information, acknowledgements, peer review information; details of author contributions and competing interests; and statements of data and code availability are available at 10.1038/s41566-025-01791-1.

## Supplementary information

Supplementary Information Supplementary Notes 1–4, Figs. 1–49, Tables 1–3 and References.

Reporting Summary

## Data Availability

All data supporting the findings of this study are available in the article and its Supplementary Information. Data are also available from the corresponding authors upon reasonable request.
