# Supplementary material for: Stabilizing high-efficiency perovskite solar cells via strategic interfacial contact engineering
Source: Nat Photonics. 2025 Nov 7;20(1):55–62. doi: 10.1038/s41566-025-01791-1 (PMC12774855; doi:10.1038/s41566-025-01791-1)
Supplement: Supplementary file 1 — Supplementary Notes 1–4, Figs. 1–49, Tables 1–3 and References. [file 41566_2025_1791_MOESM1_ESM.pdf]

nature photonics

Supplementary information https://doi.org/10.1038/s41566-025-01791-1

Stabilizing high-efficiency perovskite solar cells via strategic interfacial contact engineering

In the format provided by the authors and unedited

Table of Contents

Supplementary Notes 1 to 4

Supplementary Figures S1 to S49

Supplementary Tables S1 to S3

Supplementary References 1 to 15

Supplementary Notes

Supplementary Notes 1: Grazing-incidence wide-angle X-ray scattering (GIWAXS)

GIWAXS measurements were performed at the mySpot beamline, BESSY II.[1] The samples were measured at room temperature in reflection mode with incidence angles from 0 to 2° (0.05° interval), using a radiation energy of 9 keV (λ = 1.378 Å). The size of beam is around 50 by 50 μm^2.

Supplementary Notes 2: Transient surface photovoltage (SPV)

SPV measurements at 1 Hz were performed on encapsulated samples using 688 nm excitation laser light from a diode-pumped tunable pulse laser (Nd:YAG, EKSPLA, NT230-50, duration time of laser pulses 3-5 ns) with a spectral cleaning unit. The measurement frequency was 1 Hz, 30 averages were taken per transient, and the laser intensity (fluence of 0.010 $\mu J/cm^2$ equivalent to 1 sun) was controlled using neutral density filters. The SPV transients were measured with an oscilloscope card (Gage, CSE 1622-4GS, 200 MS/s) using in-house developed software for logarithmic readout.[2] Excitation was performed from the perovskite side.

Supplementary Notes 3: Computational details

Calculations were conducted using the CASTEP code[3], implementing density functional theory (DFT)-based first-principles methods. For the exchange-correlation potential, the generalized gradient approximation (GGA)[4] with the Perdew–Burke–Ernzerhof (PBE) formula[5] was employed, along with DFT-D corrections. The Broyden–Fletcher–Goldfarb–Shanno (BFGS) method was utilized to locate the ground state of the supercells. Convergence tolerances were set with an energy change threshold below 10-5 eV per atom, forces less than 0.02 eV $Å^{-1}$, stress below 0.05 GPa, and displacement changes under 0.001 Å. The plane-wave basis set cutoff energy was fixed at 450 eV.

Supplementary Notes 4: Calculation of hysteresis index

The hysteresis index (HI) of the PSCs was obtained by calculating the difference between forward-scan and reverse-scan efficiencies from the *J-V* curves, defined as:[6]

$$\text{Hysteresis index} = \frac{PCE_{reverse} - PCE_{forward}}{PCE_{reverse}}$$

To avoid the operational influence, a consistent scan rate and range were used to measure all *J-V* curves for forwarded and reversed scans.

Supplementary figures

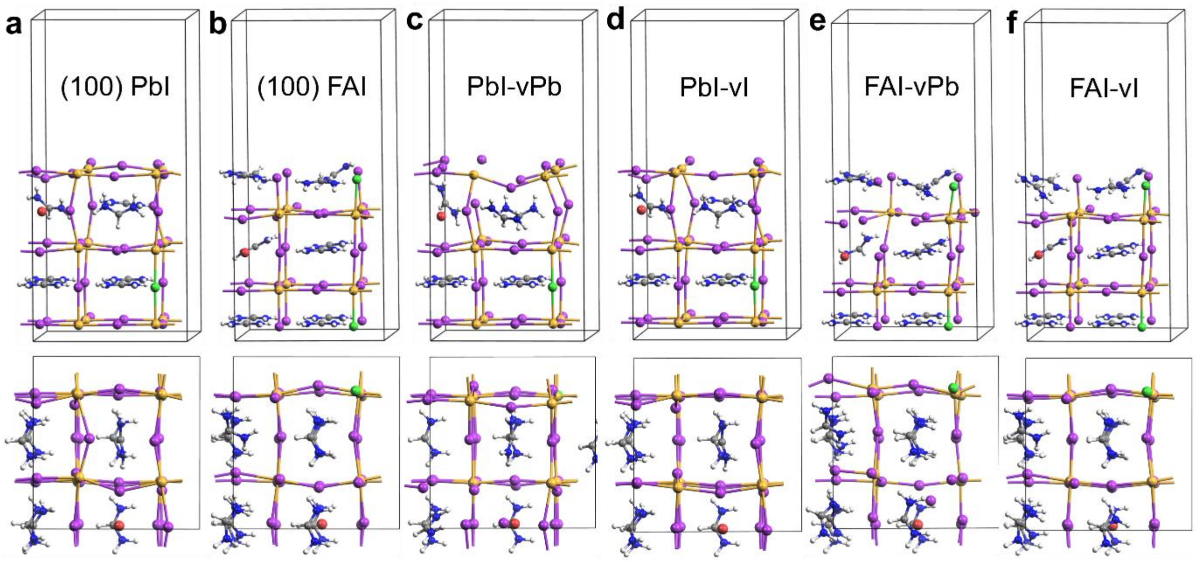

Figure S1. Perovskite configuration types: (**a**) surface PbI termination, (**b**) surface FAI termination, (**c**) Pb defect and (**d**) I defect for PbI termination, (**e**) Pb defect and (**f**) I defect for FAI termination. The vacancy defect system is abbreviated as v, and the defect forms of Pb and I are recorded as vPb and vI, respectively. Side view (upper) and top view (bottom).

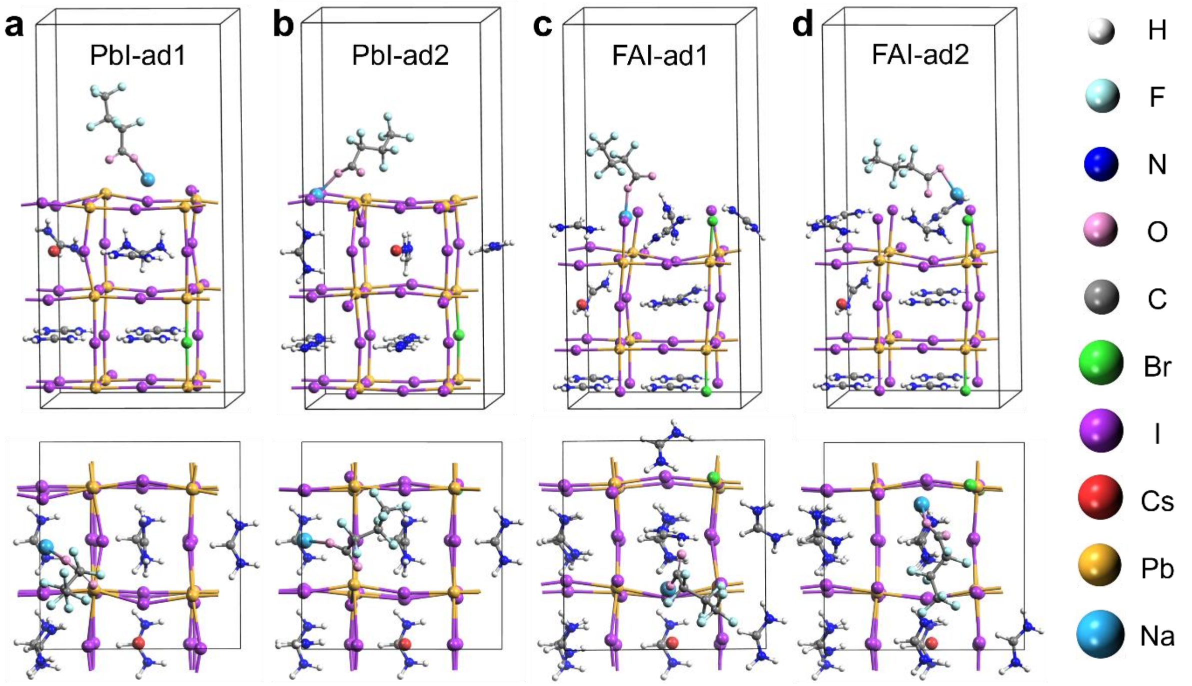

Figure S2. Perovskite surface adsorption configurations: (**a**) vertical adsorption and (**b**) horizontal adsorption on PbI-terminated perovskite surfaces; (**c**) vertical adsorption and (**d**) horizontal adsorption on FAI-terminated perovskite surfaces. The surface adsorption system is abbreviated as ad, where the initial vertical adsorption is recorded as ad1 and the initial horizontal adsorption is recorded as ad2. Side view (upper) and top view (bottom).

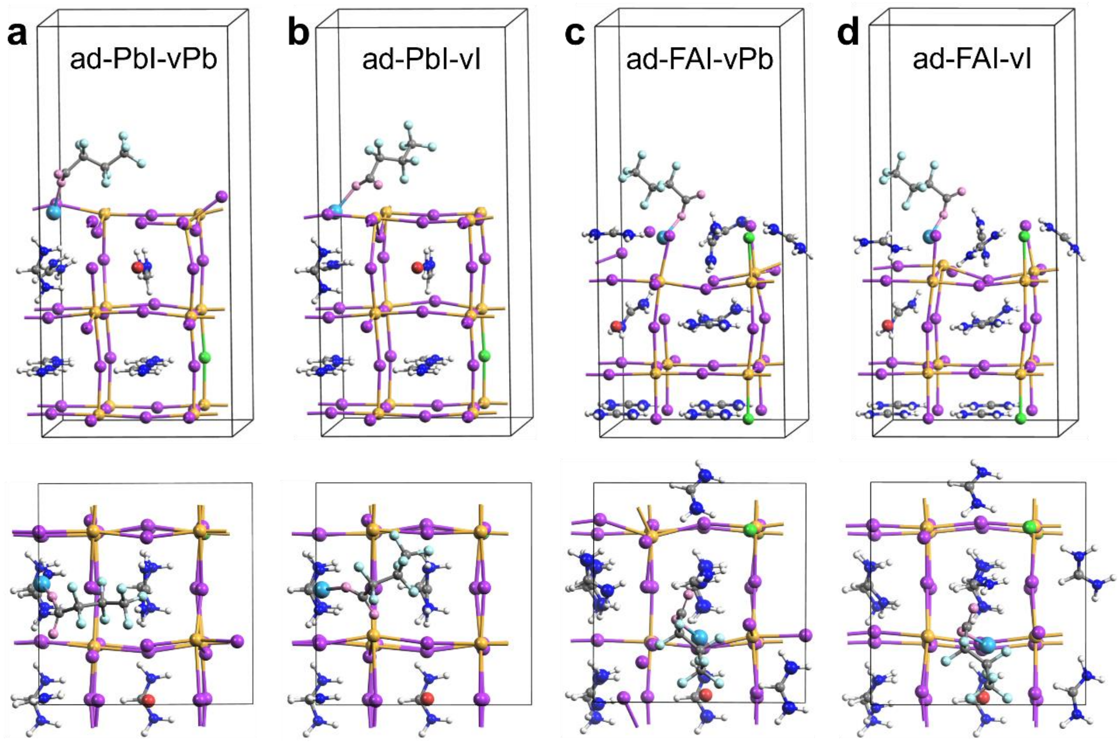

Figure S3. Perovskite surface adsorption configurations. Adsorption of SHF on PbI-terminated perovskite surfaces with (**a**) Pb vacancies and (**b**) I vacancies; SHF adsorption on FAI-terminated perovskite surfaces with (**c**) Pb vacancies and (**d**) I vacancies. The vacancy defect system is abbreviated as v, and the defect forms of Pb and I are recorded as vPb and vI, respectively. Side view (upper) and top view (bottom).

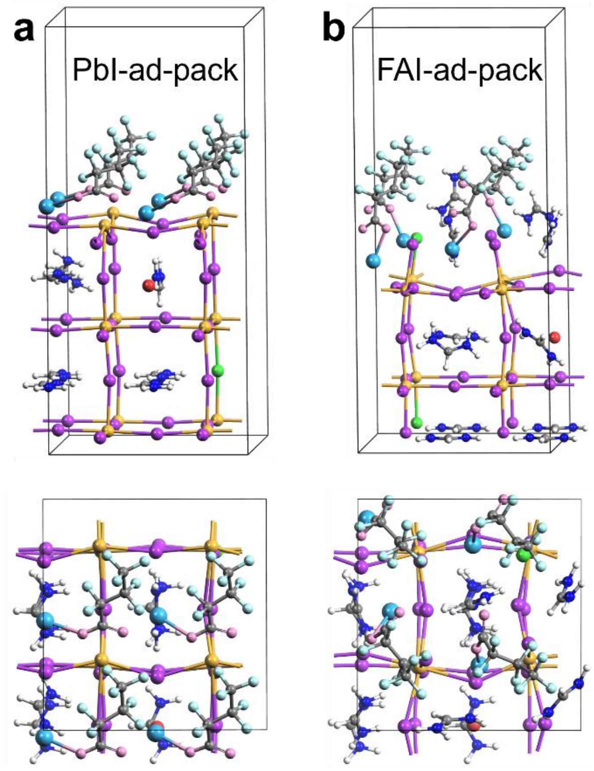

Figure S4. Packing of four SHF units on different terminated perovskite surfaces. (**a**) surface adsorption on PbI-terminated perovskite; (**b**) surface adsorption on FAI-terminated perovskite. The surface adsorption system is abbreviated as ad. Side view (upper) and top view (bottom).

DFT modeling reveals that in the surface adsorption system, the formation energy of vacancies on the PbI surface significantly increases (**Figures 1b, S1-S4**). For the FAI surface, as Pb(II) are not on the surface, the ability to form vacancies remains largely consistent with the pure surface system. Surface modification with SHF greatly enhances the stability of the PbI surface, which is beneficial for the overall stability of the system.

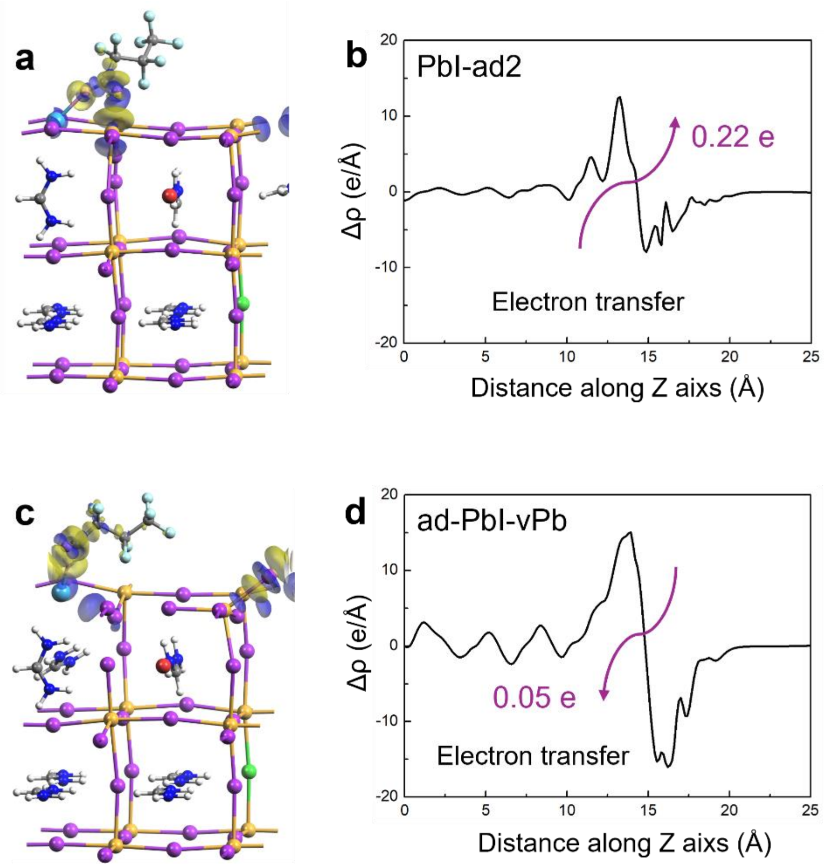

Figure S5. Electron transfer for adsorption of a single SHF. (**a**) 3D and (**b**) 1D Δρ for PbI-terminated surface of SHF-adsorbed perovskite. (**c**) 3D and (**d**) 1D Δρ for PbI-terminated surface of SHF-adsorbed perovskite with Pb vacancies. If the adsorbate gains electrons, the Y axis is abbreviated as "+", otherwise it is abbreviated as "–".

Using Mulliken charge population analysis, the electron transfer amounts for six different systems were calculated (**Figures S5-S7**). For the PbI-terminated surface, we found that the adsorption of SHF on the perovskite surface results in the adsorbate gaining electrons, with a charge transfer amount of 0.22e (**Figure S5a, b**). In the Pb defect system, 3D differential analysis reveals that the interactions between the surface and the adsorbate become complex, with multiple distinct regions of electron gain and loss. The absence of surface Pb leads to a slight net electron transfer from the adsorbate to the surface (**Figure S5c, d**). However, the formation of Pb defects alters the direction of electron transfer, resulting in a very high defect formation energy. This indicates that after SHF modifies the surface, the surface stability is enhanced, making defect formation less likely.

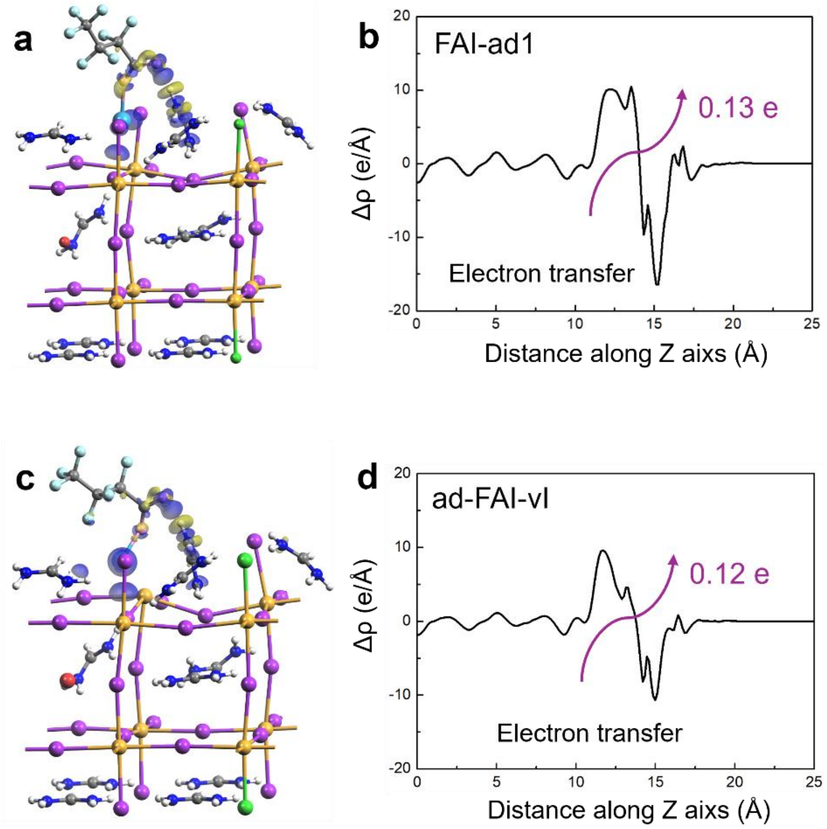

Figure S6. Electron transfer for adsorption of a single SHF. (**a**) 3D and (**b**) 1D Δρ for FAI-terminated surface of SHF-adsorbed perovskite. (**c**) 3D and (**d**) 1D Δρ for FAI-terminated surface of SHF-adsorbed perovskite with I vacancies. If the adsorbate gains electrons, the Y axis is abbreviated as "+", otherwise it is abbreviated as "–".

For the FAI-terminated surface, the formation of I defects did not significantly affect the electron transfer amount, with essentially consistent transfer amounts observed. This is corroborated by the similar differential shape distributions seen in the 3D differential analysis.

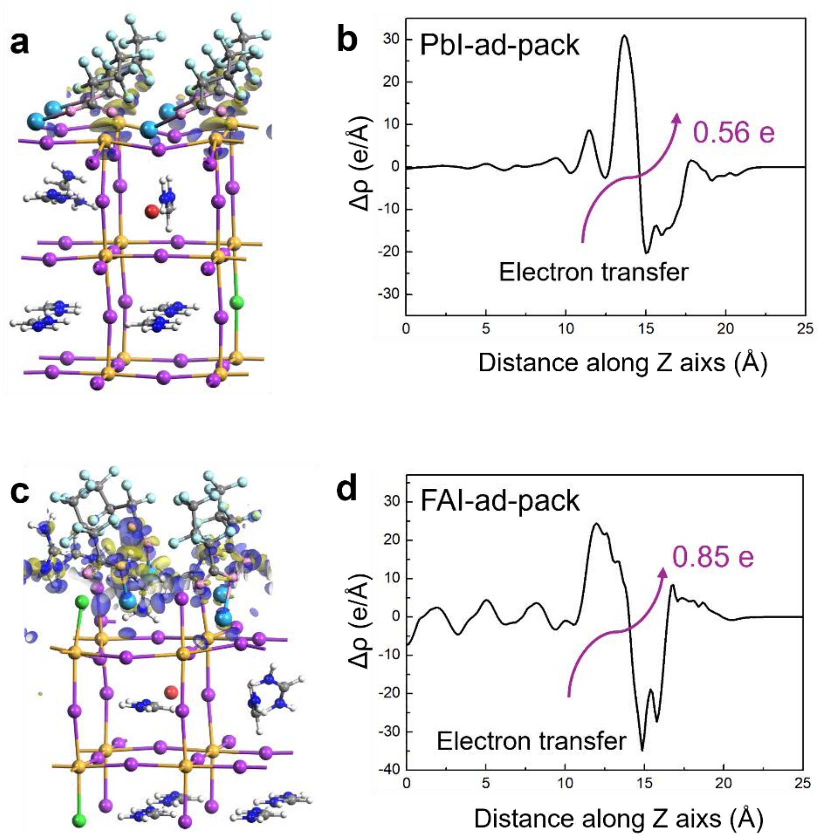

Figure S7. Electron transfer for SHF packing. (**a**) 3D and (**b**) 1D Δρ for PbI-terminated surface of SHF-stacked perovskite. (**c**) 3D and (**d**) 1D Δρ for FAI-terminated surface of SHF-stacked perovskite. If the adsorbate gains electrons, the Y axis is abbreviated as "+", otherwise it is abbreviated as "–".

For the packing systems at both the PbI and FAI terminations, the presence of more adsorbates on the surface results in a significant increase in the electron transfer amount. Furthermore, the increase in electron transfer is less pronounced for PbI termination compared to FAI termination. This difference is likely related to the inherent work function differences of the two types of terminal surfaces, the stable configurations and bonding interactions formed after adsorption of both systems.

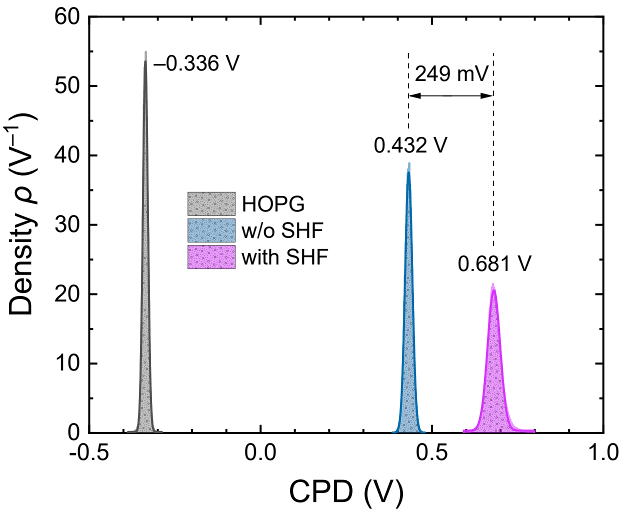

Figure S8. CPD distribution of perovskite films.

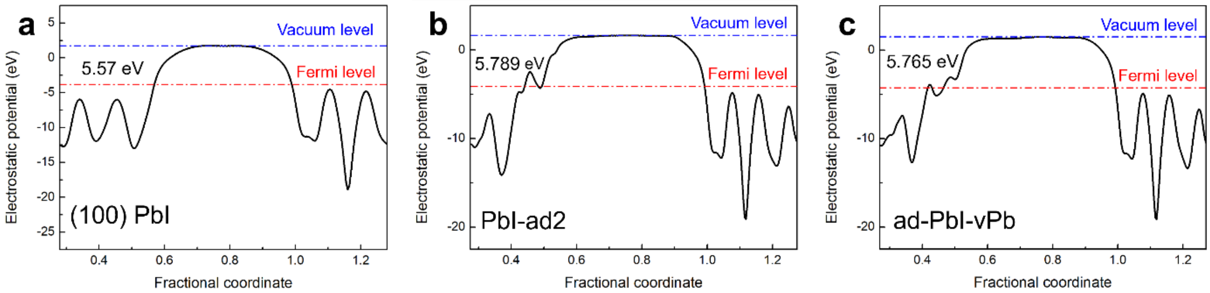

Figure S9. Calculated electrostatic potentials and work functions: (**a**) (100) plane of a PbI-terminated perovskite, (**b**) SHF adsorption on a PbI-terminated perovskite surface, (**c**) SHF adsorption on a PbI-terminated perovskite surface with Pb vacancies.

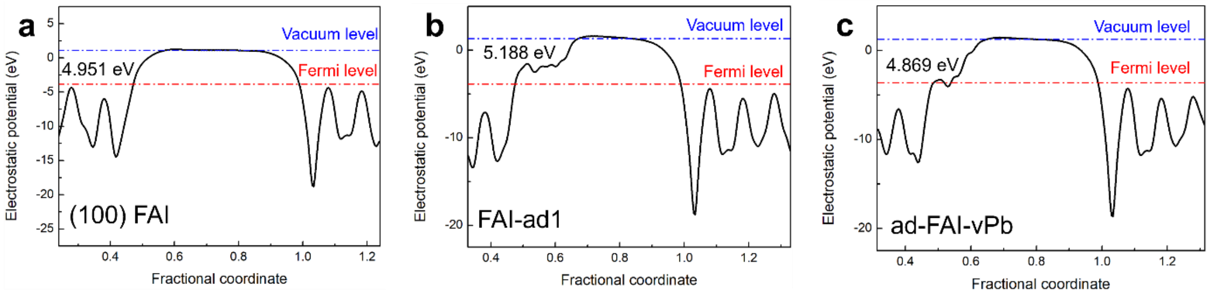

Figure S10. Calculated electrostatic potentials and work functions: (**a**) (100) plane of a FAI-terminated perovskite, (**b**) SHF adsorption on a FAI-terminated perovskite surface, (**c**) SHF adsorption on a FAI-terminated perovskite surface with Pb vacancies.

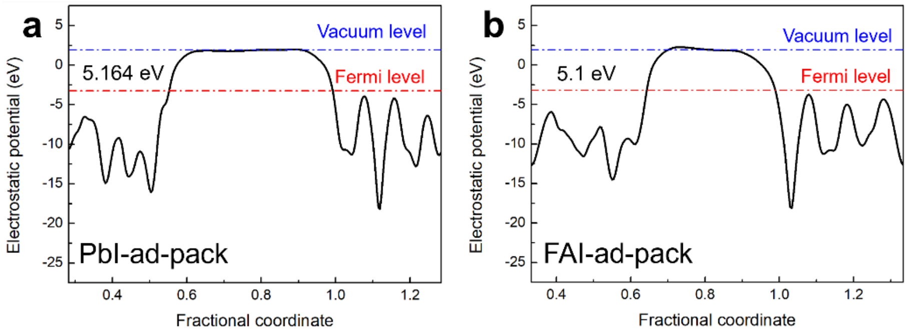

Figure S11. Calculated electrostatic potentials and work functions: packing models on (**a**) PbI-terminated and (**b**) FAI-terminated perovskite surfaces.

From the computed models (**Figures S9-S11**), the work function of the adsorption system is higher than that of the pristine surface, indicating that SHF increases the work function. For the PbI-terminated adsorption system, the work function slightly decreases but remains higher than that of the pristine surface. In the case of the surface packing system, the overall trend shows a work function lower than that of the pristine surface. This decrease is likely due to the high coverage of SHF, which results in a relatively lower work function, and implies that the coverage of SHF on the PbI-terminated surface needs to be adjusted to achieve the maximum shift in work function.

An overall increase in the work function for the FAI-terminated adsorption system was observed, from the pure FAI surface to the adsorbed surface and then to the packing surface. This suggests, like the PbI-terminated case, that SHF increases the surface work function. However, with excessive adsorbate coverage, the work function tends to decrease. Although the decline in the work function for the FAI-terminated accumulation system is not as pronounced as for that of the PbI-terminated system, it indicates that appropriate surface coverage is one of the key factors in tuning the surface work function to its maximum.

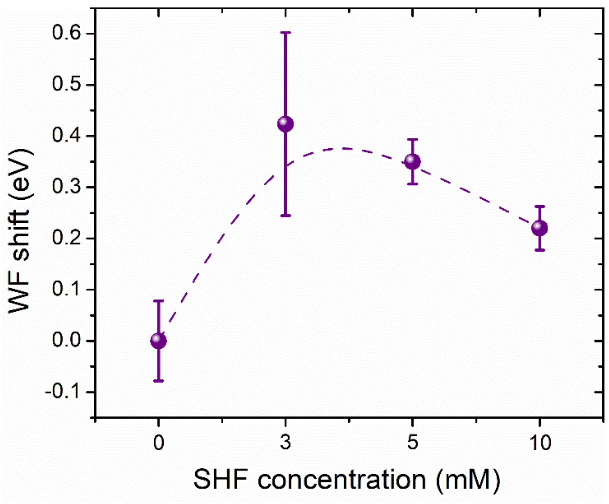

Figure S12. Surface WF shift obtained from Kelvin probe measurements of perovskites treated with different concentrations of SHF (n=3 independent films per condition, error bars represent standard deviation).

We subsequently compared surface WF shifts with different concentrations of SHF treatment, showing an increase in WF of 400 mV on the perovskite surface using 3 mM SHF (**Figure S12**), which is used for subsequent perovskite processing.

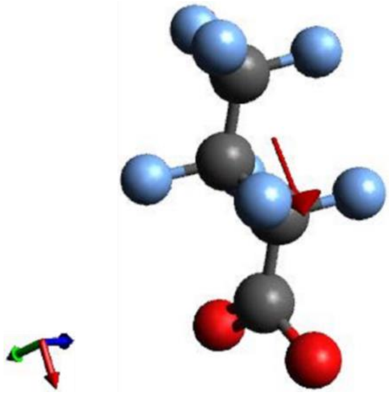

Figure S13. Structure and polarity orientation of the anion in SHF.

These positive dipoles toward the perovskite, generated by the localized charges and the strongly electronegative fluorinated tail of SHF, would lead to charge depletion around the coordinated Pb atoms in the perovskite surface while increasing electron density around the SHF molecule. This redistribution of charges would result in an increased WF value of the perovskite, as well as increasing the effective built-in potential of the full device stack, potentially boosting charge extraction under operating conditions. In other words, the observed increase in surface work function after SHF treatment reflects a dipole-induced carrier redistribution at the perovskite interface (discussed in the photovoltaic performance section). The interfacial dipole, physically established prior to active carrier transfer, functionally enhances the PSC's built-in potential, increasing the range of applied forward bases for which there is a driving force for electron extraction. Under equilibrium measurement conditions, such as dark-state KPFM, this charge redistribution leads to a detectable increase in work function, which represents a dipole-induced charge transfer.

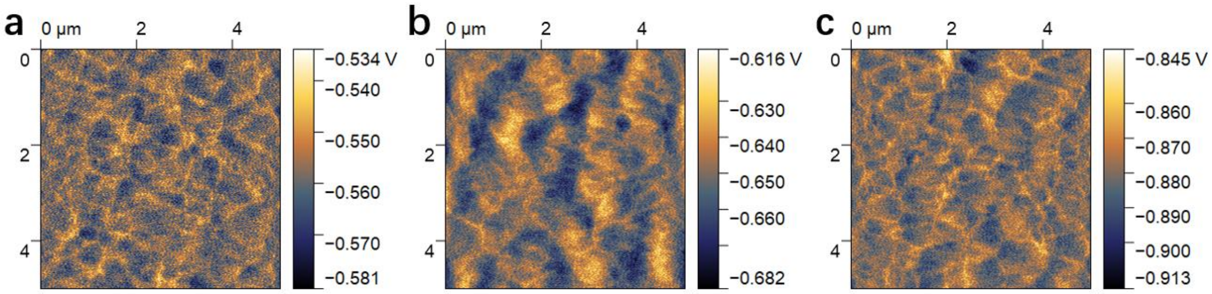

Fig. S14. KPFM maps of perovskites: (**a**) control, (**b**) NaOAc, and (**c**) SHF (additional measurement applied for this batch). Scale bars: 5 µm. Colour scale represents surface potential (V).

The difference in the surface potential can be attributed to the molecular structure: NaOAc induces a weak interfacial dipole, while SHF, bearing a strong molecular dipole, effectively modulates the interfacial energetics.

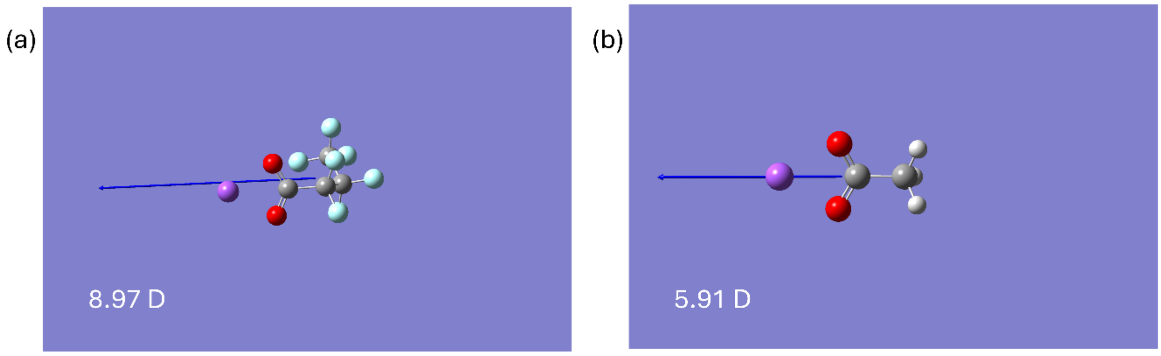

Figure S15. Calculated dipole moments of (**a**) SHF and (**b**) NaOAc (without F-substituted tail) molecules.

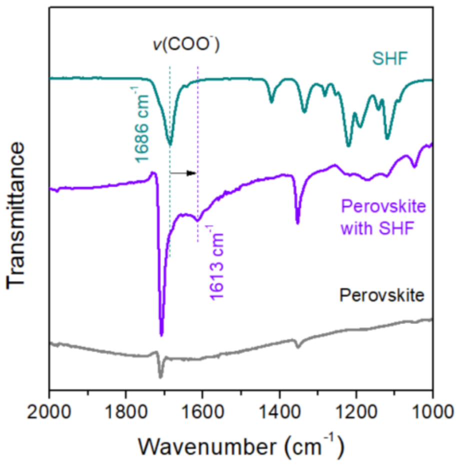

Figure S16. FTIR of SHF, untreated and treated perovskite films.

Fourier transform infrared (FTIR) spectroscopy was used to probe the interactions between SHF and the perovskite. FTIR spectra reveal bond formations (**Figure S16**). For SHF, the peak located at 1686 cm^{-1} is assigned to the COO^{-} bond stretching vibration. The COO^{-} peak shifts to a much lower wavenumber of 1613 cm^{-1} in the SHF-treated perovskite film, indicating a strong interaction between the COO^{-} group and Pb(II) ion.

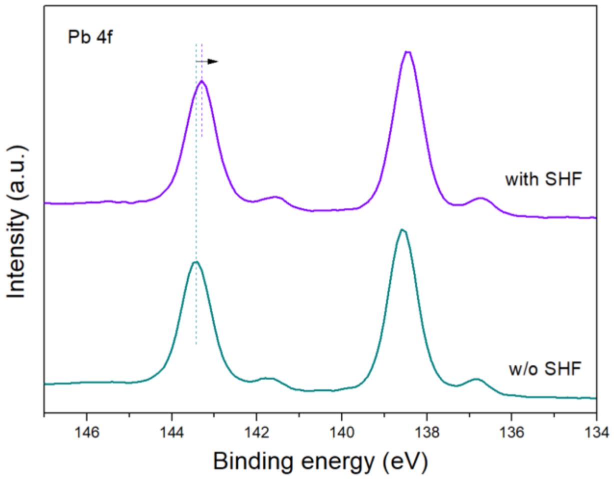

Figure S17. Pb 4f XPS spectra of perovskite films with and without SHF treatment.

Upon SHF treatment, a noticeable shift in the Pb 4f binding energy (EB) to lower values was observed in the perovskite films (**Figure S17**). This downshift is attributed to the strong coordination between SHF and Pb(II) ions in the perovskite, which modified the electronic environment of Pb.

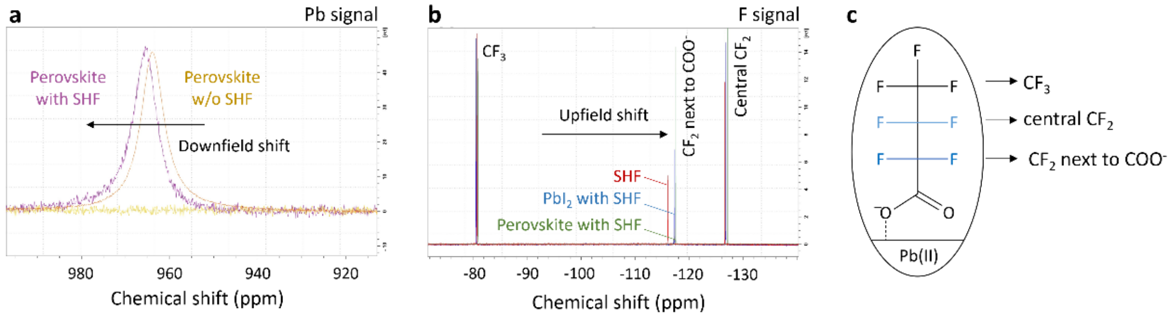

Figure S18. Solution-state ^{207}Pb and ^{19}F NMR spectra of the perovskite precursor solution without and with SHF: (**a**) Pb signal and (**b**) F signal, following (**c**) a schematic of SHF interacting with perovskite.

The shift of the peaks in the ^{207}Pb NMR and ^{19}F NMR spectra (CF_3 < central CF_2 < CF_2 next to COO^-) indicates that the carboxylate group coordinates to the perovskite's Pb(II), as shown in **Figure S18**. This supports that SHF is able to anchor to the perovskite surface via COO...Pb bonds.[7]

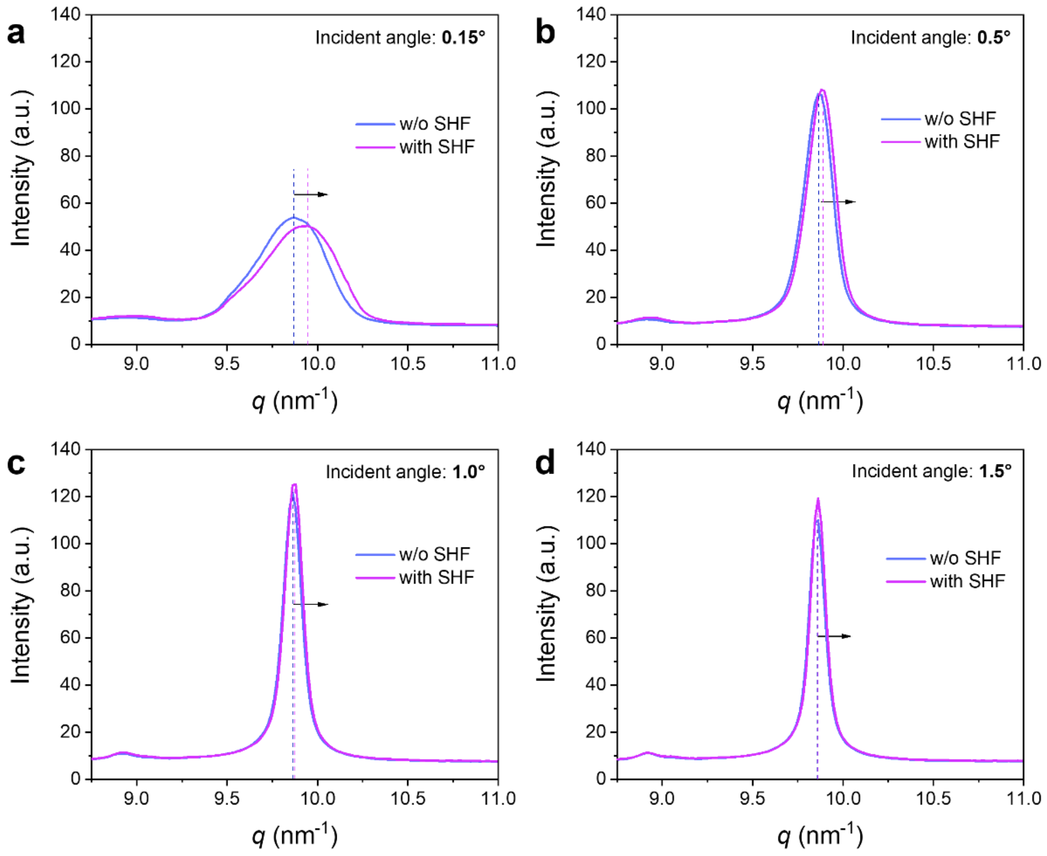

Figure S19. Incident angle dependent 1D GIWAXS curves of the untreated and SHF-treated perovskite films as a function of the scatting vector: incident angles of (**a**) 0.15°, (**b**) 0.5°, (**c**) 1.0° and (**d**) 1.5°.

Observed contraction may result from lattice adjustment caused by SHF migrating to grain boundaries at the film's surface, potentially driven by a molecular squeezing effect.

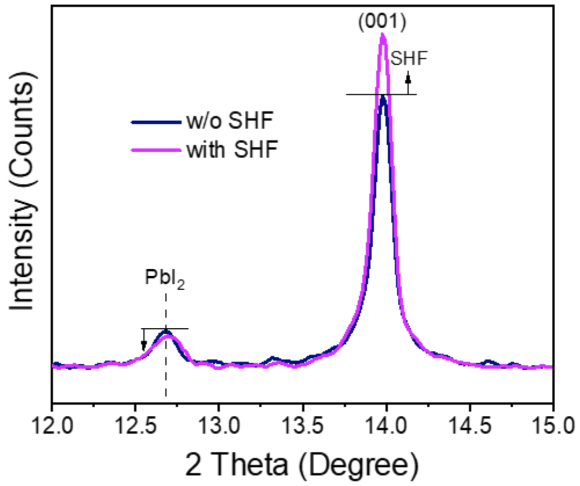

Figure S20. XRD patterns of the untreated and SHF-treated perovskite films.

The intensity of the peak at 13.98° corresponding to the (001) plane of perovskite is enhanced for the SHF-treated sample. The peak of PbI_2 has a slightly lower intensity in the SHF-treated sample, suggesting that SHF interacts with excess PbI_2, providing a more stable surface environment.[8-10]

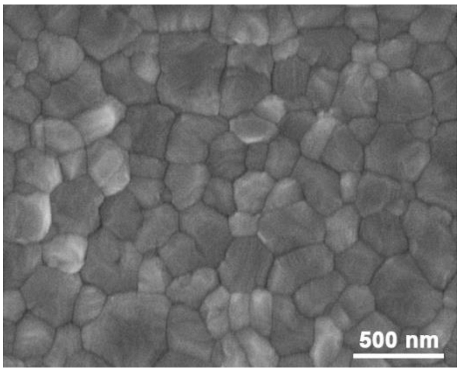

Figure S21. SEM images of the untreated perovskite films.

Compared to control film, the SHF-treated film exhibits a smoother surface, presumably due to interactions between the components in the perovskite and SHF that form during the post-treatment process.

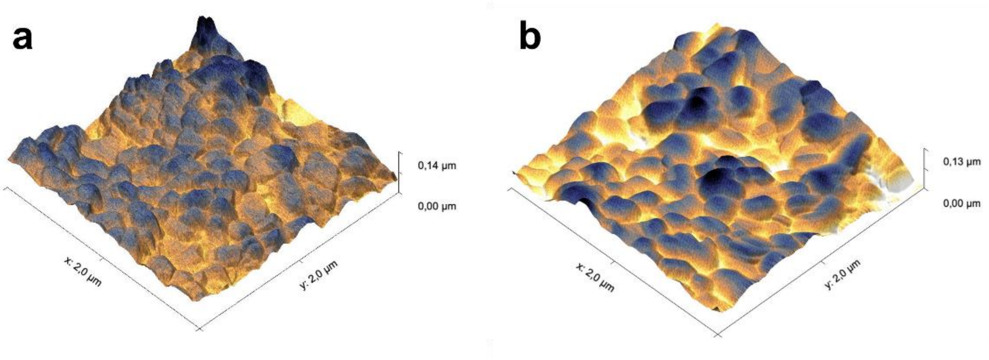

Figure S22. AFM surface morphology of the untreated (**a**) and SHF-treated (**b**) perovskite films.

Three-dimensional AFM topography shows the surface morphology in perovskite film (area of 2 µm × 2 µm, see **Figure S22**). The reduced film surface roughness upon SHF treatment is reflected by the root mean square (RMS) values from 21.1 nm to 18.9 nm, which may provide a better interface contact with the ESC. Scale bars: 2 µm. Colour scale represents surface height (nm).

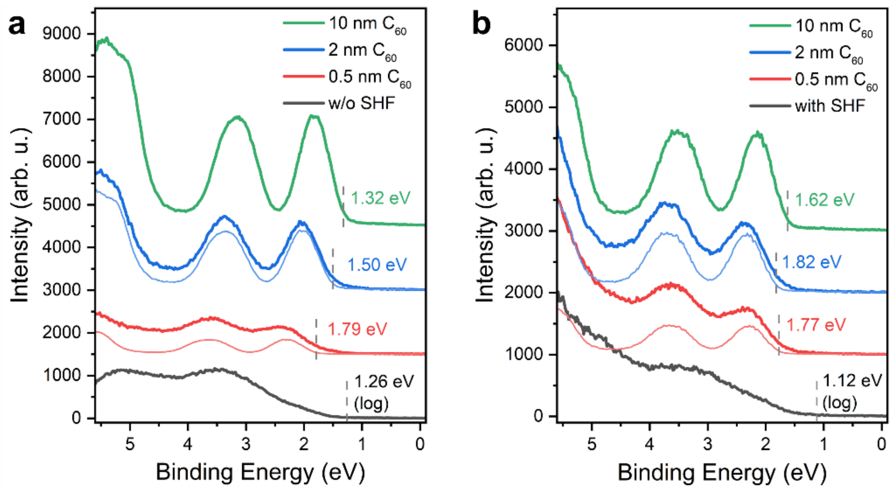

Figure S23. UPS spectra of C_{60} with different deposition thicknesses on untreated (**a**) and SHF-treated (**b**) perovskite films.

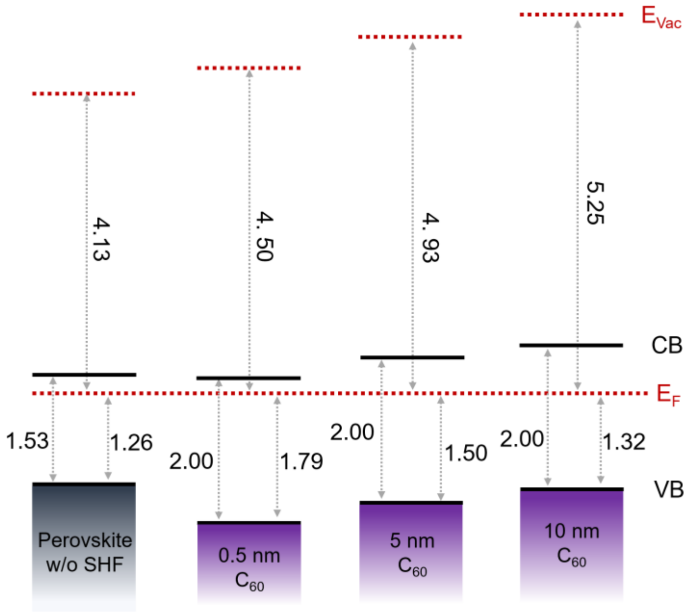

Figure S24. Energy-level diagram for untreated perovskite/C_{60}.

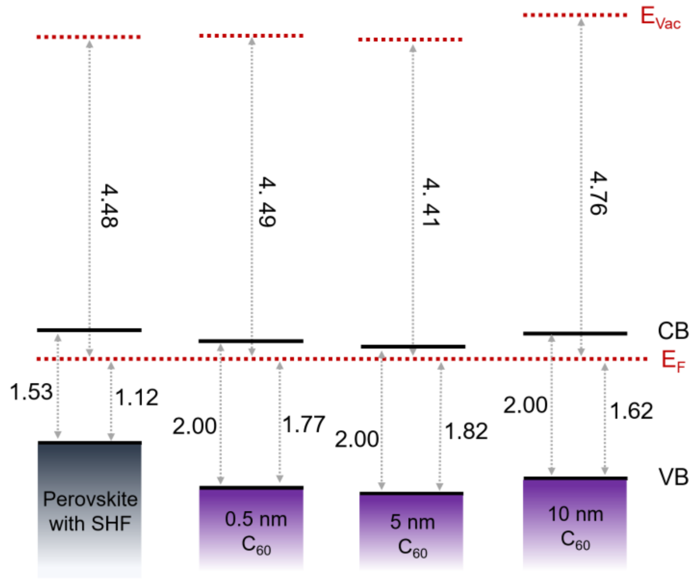

Figure S25. Energy-level diagram for SHF-treated perovskite/C_{60}.

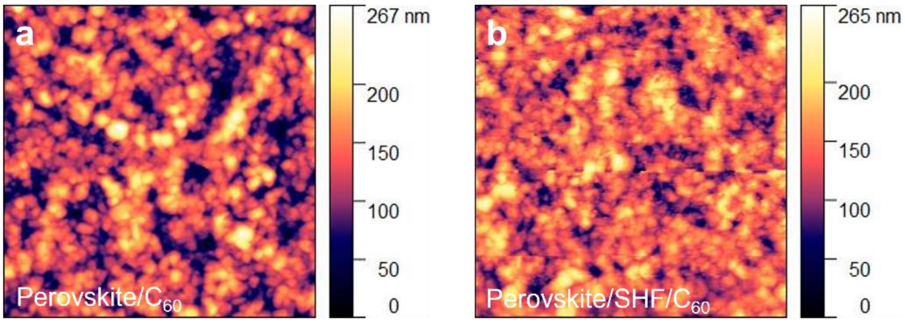

Figure S26. AFM images at a 10 × 10 μm^2 scale of (**a**) perovskite/C_{60} and (**b**) perovskite/SHF/C_{60} (2 nm C_{60} deposited). Scale bars: 10 μm. Colour scale represents surface height (nm).

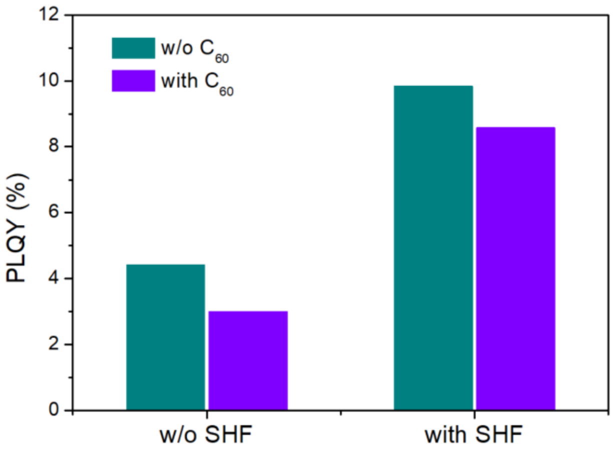

Figure S27. PLQY of perovskite, perovskite/SHF, perovskite/C_{60} and perovskite/SHF/C_{60} samples.

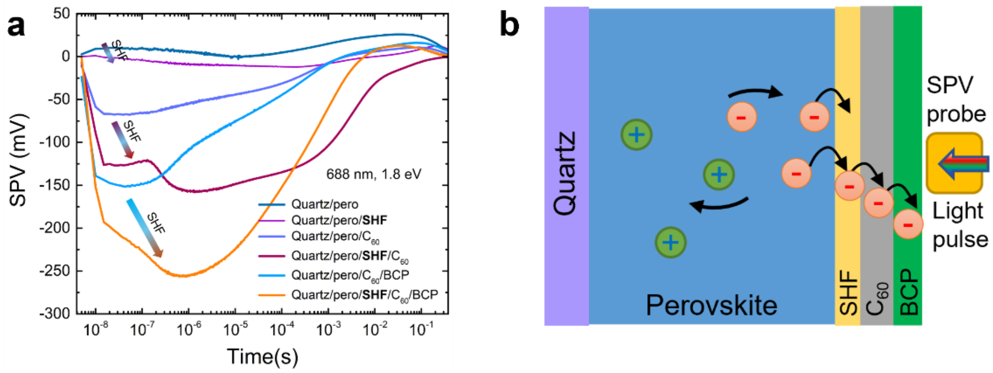

Figure S28. (**a**) Transient SPV of perovskite, perovskite/SHF, perovskite/C_{60}, perovskite/SHF/C_{60}, perovskite/C_{60}/BCP and perovskite/SHF/C_{60}/BCP samples on quartz measured at an excitation of 688 nm (1.8 eV). (**b**) Illustration of the charge extraction and recombination model that describes carrier transport.

Charge extraction was examined in substrate/perovskite/ESC samples by adding each layer to the ESC side in steps. In a p-i-n solar cell structure, the extraction of electrons towards the ESC results in a negative SPV signal, observed as a rise in the SPV signal. The faster rise indicates a higher extraction rate.

The trSPV signal amplitudes for the sample interfaces treated with SHF increase noticeably compared to the interface without SHF.

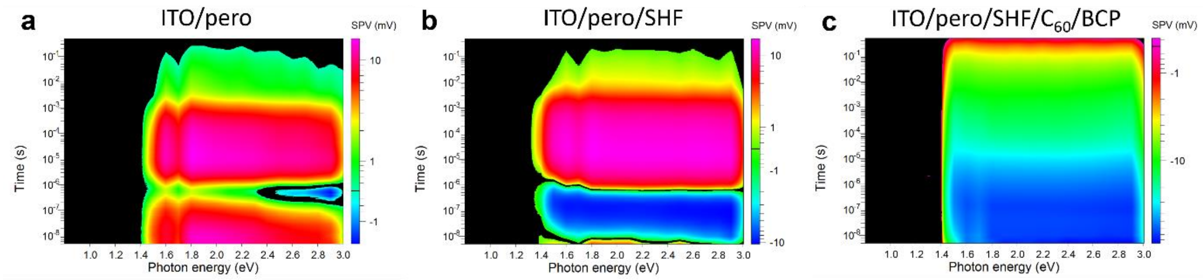

Figure S29. Contour plots of the transient SPV illuminated by different light energies for (**a**) ITO/perovskite, (**b**) ITO/perovskite/SHF and (**C**) ITO/perovskite/SHF/C_{60}/BCP.

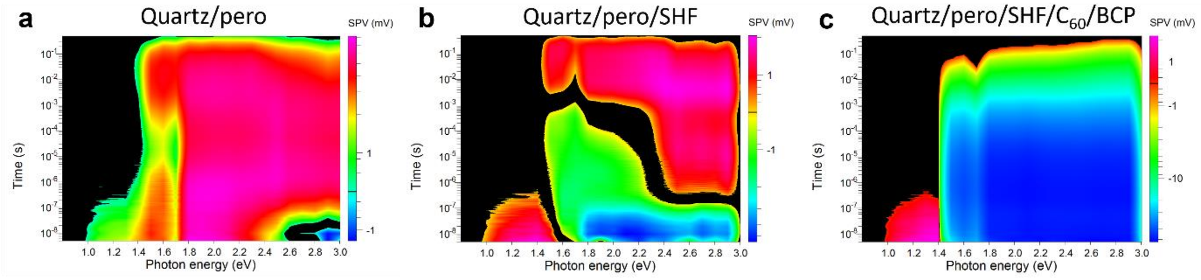

Figure S30. Contour plots of the transient SPV illuminated by different light energies for (**a**) quartz/perovskite, (**b**) quartz/perovskite/SHF and (**c**) quartz/perovskite/SHF/C_{60}/BCP.

The amplitude and decay of the SPV signal are influenced by poor selectivity and charge losses. In a p-i-n solar cell structure, the extraction of electrons to the ETL results in a negative SPV signal, observed as a rise in the SPV signal. A faster rise corresponds to a higher extraction rate (Re = n * Ke). A positive SPV amplitude can result from inferior charge selectivity of the ETL and hole trapping at the interface.

The main difference between the results for quartz and ITO substrates was the detection of additional deep defect absorption starting near 1.0 eV by trSPV (**Figures S29** and **S30**), which was not observed with ITO. Notably, the more defective quartz/perovskite/SHF/C_{60}/BCP sample showed trSPV signals nearly comparable to the less defective ITO/perovskite/SHF/C_{60}/BCP sample (ratio 0.95:1). Moreover, the amplitude for quartz/perovskite/C_{60} is much lower than ITO/perovskite/C_{60} (ratio 0.7:1). These findings underscore the ability of SHF to enhance electron extraction and passivation.

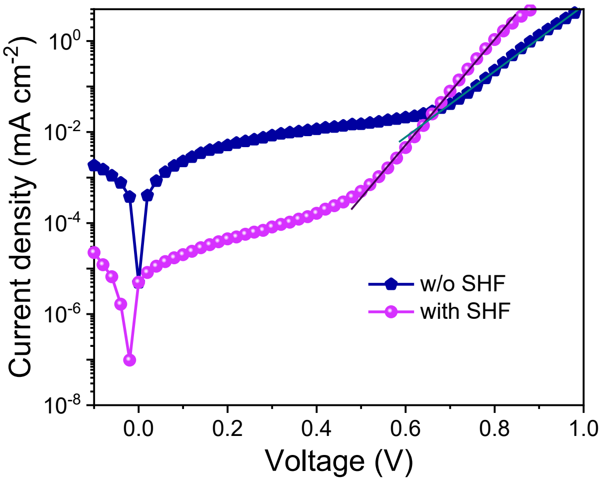

Figure S31. *J-V* curves of the control and SHF-treated devices under dark conditions.

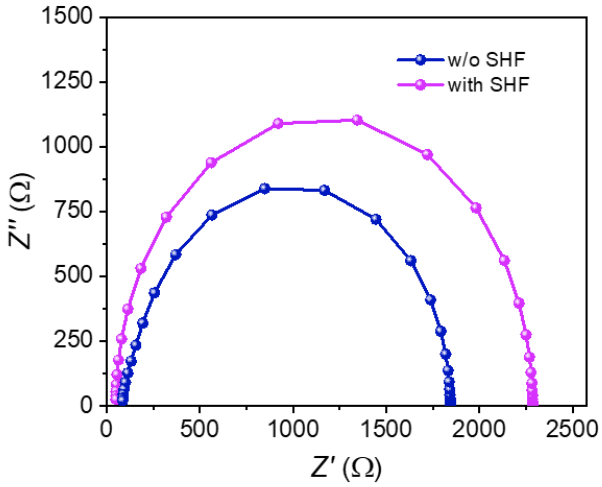

Figure S32. EIS (unfitted) of the control and SHF-treated devices in the dark.

The dark current of the SHF-treated device is significantly lower than that of the control device (about two orders of magnitude reduction), indicating that SHF treatment greatly suppresses current leakage. It is considered that the photogenerated carriers are effectively separated rather than recombined during the photoelectric conversion.[11] Simultaneously, a larger slope was observed, corresponding to lower ideality factors and higher injected current in the bias region after 0.7 V, illustrative of a lower injection barrier in the SHF-treated device.[11,12] In addition, the SHF-treated device is more resistant to recombination compared to control device, evidenced from EIS.[13,14]

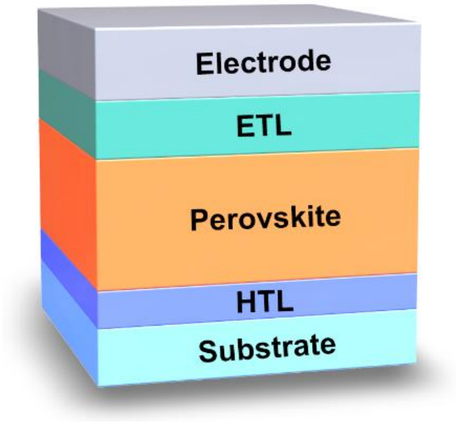

Figure S33. Architecture of the inverted p-i-n PSCs, comprising (from the bottom) conductive substrate/SAM CbzNaph/perovskite/C_{60}/BCP/Ag (SnO_2 is used to replace BCP in devices for stability testing).

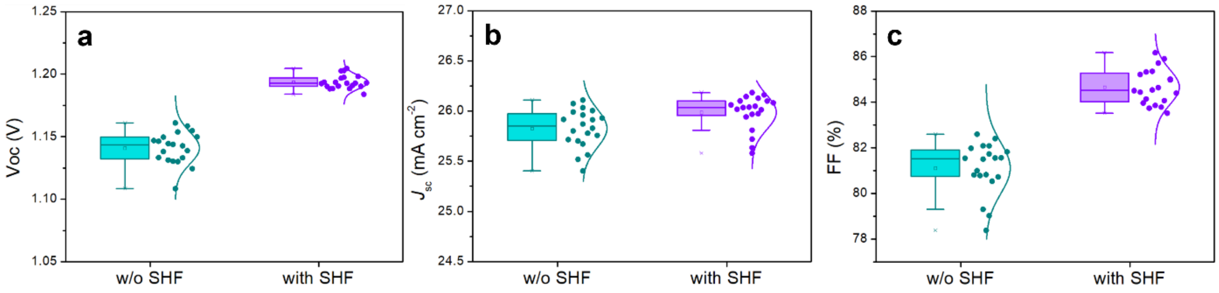

Figure S34. Statistical box charts of (**a**) V_{oc}, (**b**) J_{sc} and (**c**) FF distribution for control and SHF-treated PSCs. Twenty independently fabricated devices per condition (n=20 for each group, representing independent devices). The boxes show the interquartile range, the center line indicates the median, and the whiskers represent the 5th to 95th percentiles.

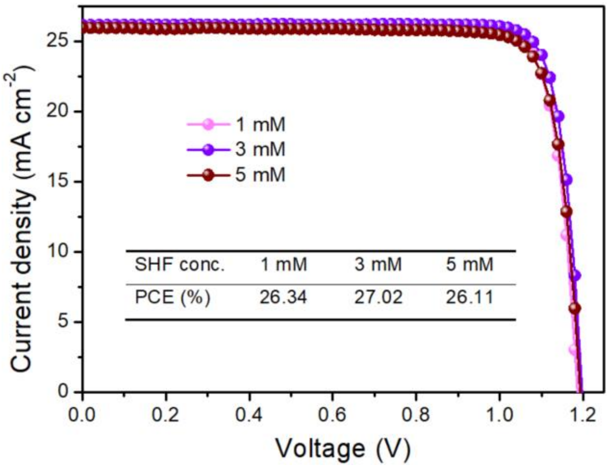

Figure S35. *J-V* curves of devices incorporating perovskites treated with different concentrations of SHF (1, 3 and 5 mM).

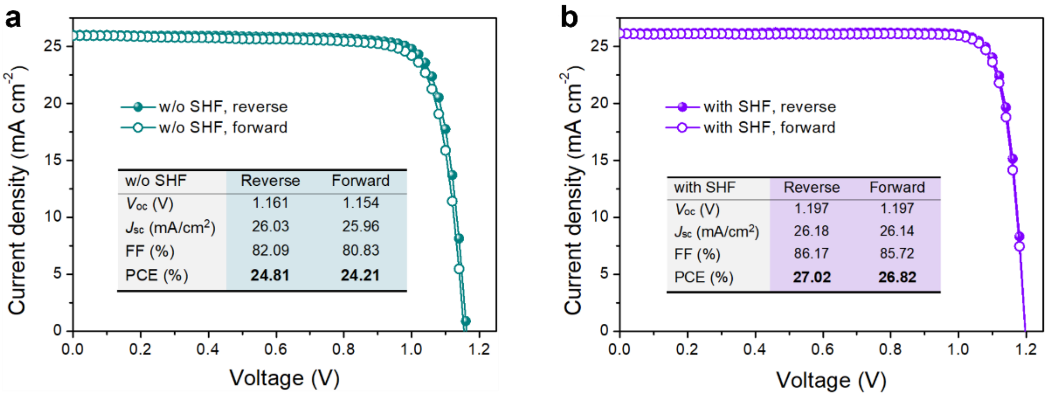

w/o SHF	Reverse	Forward
V_{oc} (V)	1.161	1.154
J_{sc} (mA/cm^2)	26.03	25.96
FF (%)	82.09	80.83
PCE (%)	**24.81**	**24.21**

with SHF	Reverse	Forward
V_{oc} (V)	1.197	1.197
J_{sc} (mA/cm^2)	26.18	26.14
FF (%)	86.17	85.72
PCE (%)	**27.02**	**26.82**

Figure S36. *J-V* curves with the reverse and forward scans for devices based on (**a**) control and (**b**) SHF-treated perovskites.

Compared to the control device, *J-V* hysteresis in the treated device is reduced when measured under forward and reverse scans (**Figure S36** and **Table S2**), which facilitates charge collection and reduces interfacial charge accumulation.

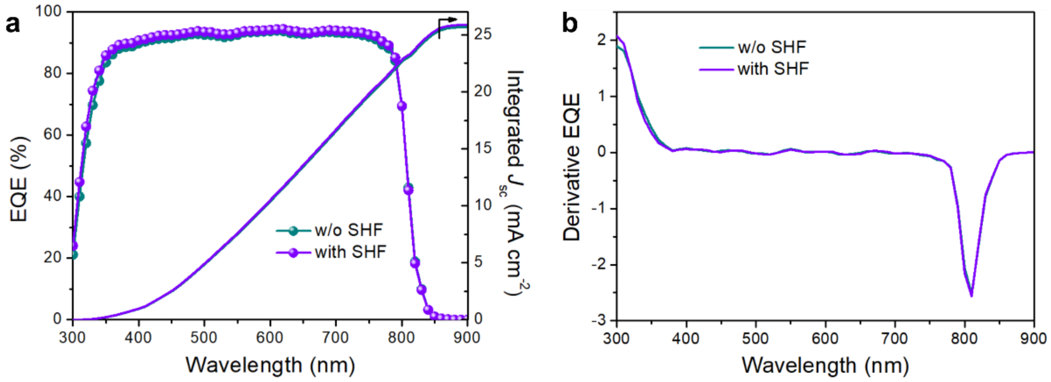

Figure S37. (**a**) EQE and integrated current densities of control and SHF-treated PSCs and (**b**) their derivative EQE spectra.

The integrated J_{sc} values from the EQE spectra are 25.7 mA/cm^2 and 25.9 mA/cm^2 for the untreated and SHF-treated PSCs (**Figure S37a**), respectively, matching well with the values acquired from the *J-V* results (<1.3% discrepancy). The bandgap of both perovskites was determined as ~1.53 eV from the derivative EQE spectra (**Figure S37b**), suggesting an unchanged optical bandgap.

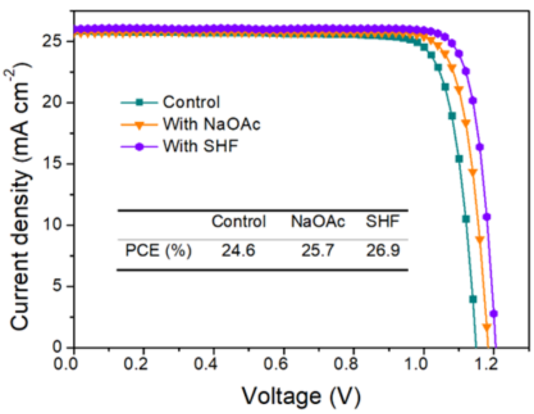

Figure S38. *J-V* curves of devices treated with NaOAc and SHF, as well as the untreated control (within one batch).

福建省计量科学研究院
FUJIAN METROLOGY INSTITUTE
（国家光伏产业计量测试中心）
National PV Industry Measurement and Testing Center

24Q3-00240

检测结果/说明：
Results of Test and additional explanation

1. Standard Test Condition (STC): Total Irradiance: 1000 W/m^2
Temperature: 25.0 °C
Spectral Distribution: AM1.5G

2. Measurement Data and I-V/P-V Curves under STC

Forward Scan

I_{sc} (mA)	V_{oc} (V)	I_{MPP} (mA)	V_{MPP} (V)	P_{MPP} (mW)	FF (%)	A (cm^2)
2.079	1.204	1.957	1.055	2.065	82.50	0.0782

Reverse Scan

I_{sc} (mA)	V_{oc} (V)	I_{MPP} (mA)	V_{MPP} (V)	P_{MPP} (mW)	FF (%)	A (cm^2)
2.076	1.207	1.987	1.061	2.108	84.13	0.0782

Mismatch factor: 0.9981

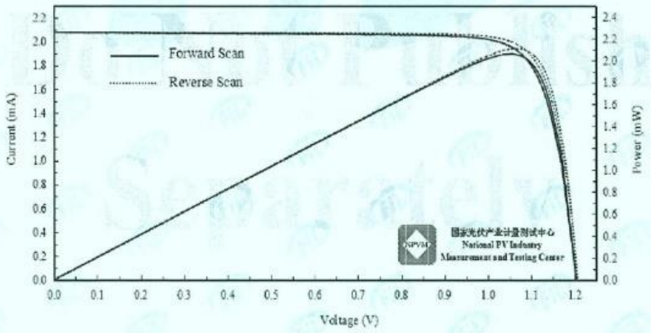

Figure 1. I-V and P-V characteristic curves of the measured sample under STC

检测报告续页专用
Continued page of test report

第 3 页/共 6 页
Page of Pages

Figure S39. Independent certification of the champion SHF-treated PSC under standard measurement conditions (STC) by an accredited Fujian Metrology Institute (National PV Industry Measurement and Testing Center).

福建省计量科学研究院
FUJIAN METROLOGY INSTITUTE
（国家光伏产业计量测试中心）
National PV Industry Measurement and Testing Center

报告编号： 24Q3-00240
Report No.

检测结果/说明：
Results of Test and additional explanation.

3.Measurement Data and Curves for MPPT under STC

η (%)	26.61
P_{MPP} (mW)	2.081
I_{MPP} (mA)	1.986
V_{MPP} (V)	1.048

Note: Measurement data for MPPT under STC in the above table was the mean value acquired during the final 30 seconds of the 300 seconds test

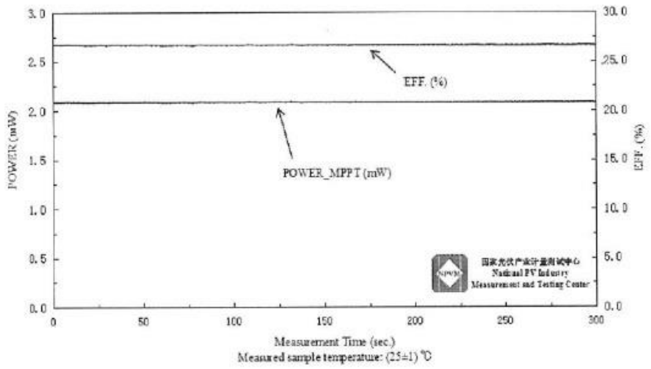

Figure 2. Measurement curves of the measured sample for MPPT

检测报告续页专用
Continued page of test report

第 4 页/共 6 页
Page of Pages

Figure S40. Certified stabilized PCE of SHF-treated (target) device under MPP tracking.

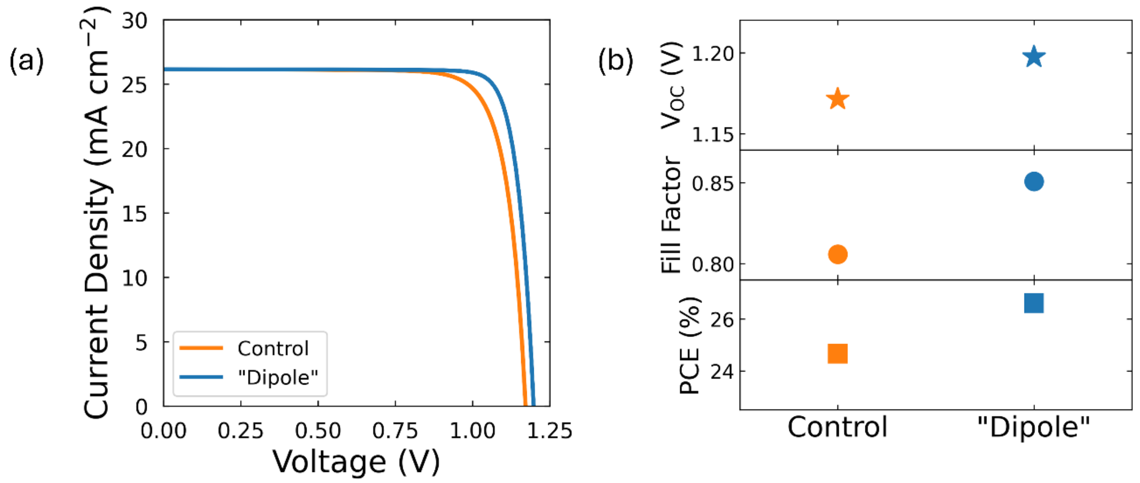

Figure S41. (**a**) Simulated current density-voltage (*J-V*) curves of the control device and the device treated with SHF ("dipole"). (**b**) Summary of the *J-V* parameters extracted from (**a**).

To gain further insight into influence of the SHF treatment on photovoltaic performance, the devices were simulated using Driftfusion, a software package that solves one dimensional drift-diffusion equations for a stack of semiconductor layers while accounting for the influence of mobile ionic species which are present in metal halide perovskites (simulation parameters given in **Table S3**). The effect of the dipole induced by SHF was incorporated as a vacuum level shift across the interface, raising the energy levels of the C_{60} layer and the metal cathode by 0.2 eV compared to the reference device, resulting in a corresponding 0.2 eV increase in the effective built-in potential (the vacuum level shift was estimated from the data in **Figures S24-S25**). These increases in performance due to the interfacial dipole arise because the reduced conduction band offset (and increased built-in potential) affects the distribution of electronic carriers in the perovskite.

The observed differences between the experimental and simulated *J-V* curves stem from uncertainties in several model parameters that are not precisely known. Such variations are common in device modeling and do not compromise the validity of the qualitative conclusions drawn from the simulation.

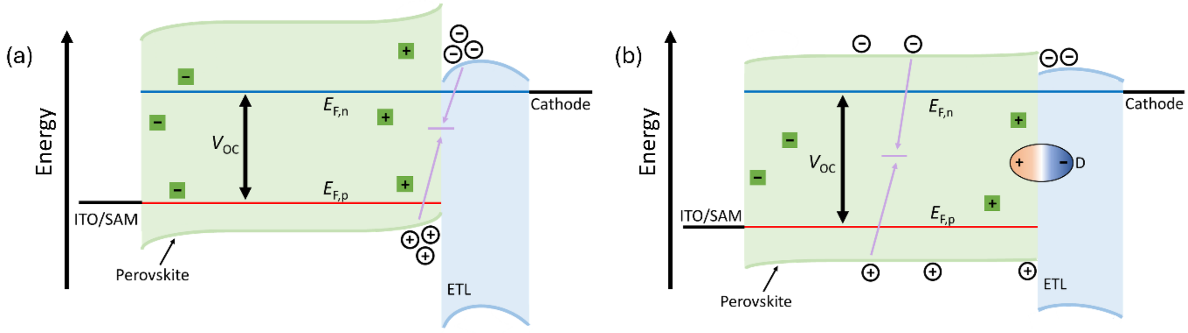

Figure S42. Schematic band diagrams under open-circuit conditions showing (**a**) the control device and (**b**) the device treated with SHF.

The positive and negative charges in white circles represent holes and electrons, respectively, while the positive and negative charges in green squares represent the accumulation and depletion of mobile ionic charge. Due to the lower built-in potential of the control device, there is a greater hole accumulation at the perovskite/C_{60} interface at a given applied voltage. This increases the rate of nonradiative recombination mediated by interfacial trap states (or trap states located at the perovskite surface), resulting in a lower V_{OC} and a fill factor. When the SHF is present, this nonradiative recombination pathway becomes less significant due to there being less hole accumulation at the perovskite/C_{60} interface and, for the parameters used herein, we find that the device's performance is limited by bulk recombination pathways, as indicated in (**b**). These results indicate that the introduction of a dipole alone is sufficient to increase device performance irrespective of any interfacial recombination centers that the SHF may passivate.

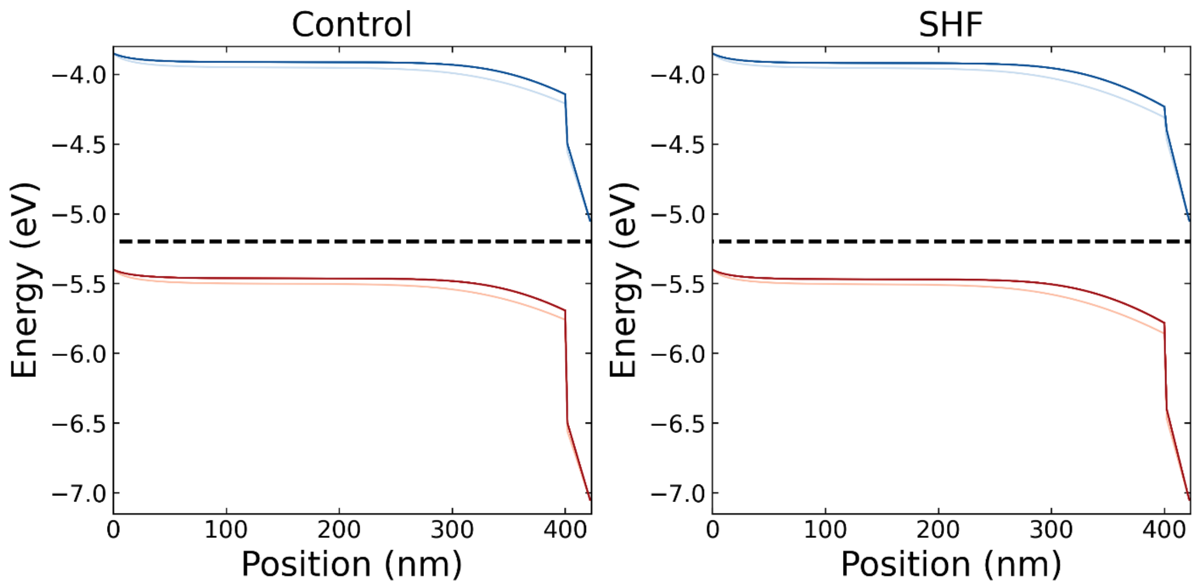

Figure S43. Drift-diffusion simulation results showing energy level profiles as a function of perovskite Fermi level variation for the Control (left) and SHF (right) devices.

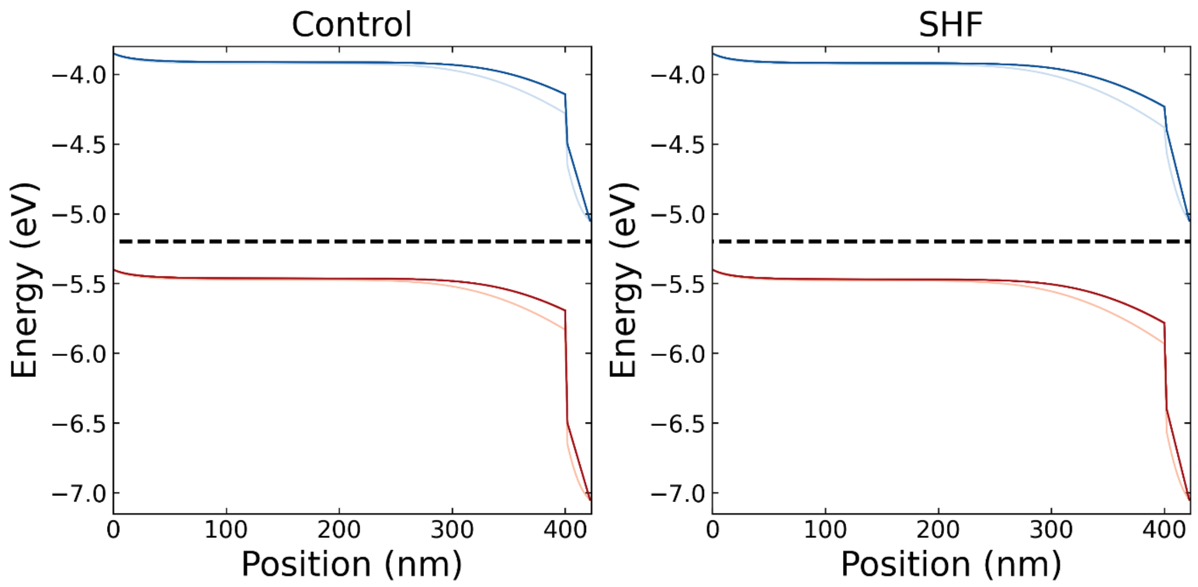

Figure S44. Simulated energy profiles with varying C_{60} Fermi levels for the Control (left) and SHF (right) devices.

To further examine the impact of Fermi level variations, we carried out simulations by systematically varying the Fermi levels of both the perovskite and C_{60} layers in complete device structures. The results show that the energy band profiles remain nearly unchanged unless the Fermi level approaches within ~150 meV of the conduction band edge. This indicates that moderate shifts in Fermi levels, such as those observed in our UPS and Kelvin probe measurements, have negligible influence on the device energetics and performance.

These findings support the view that the SHF-induced dipole, rather than bulk doping effects, is primarily responsible for the observed energetic modulation. Moreover, under typical defect densities, the influence of such Fermi level shifts becomes even less significant.

The results show that only extreme shifts near the conduction band edge led to noticeable changes. This confirms that the moderate Fermi level variations observed experimentally have negligible influence on device energetics, and that the SHF-induced dipole is the dominant factor in modifying interfacial energy alignment.

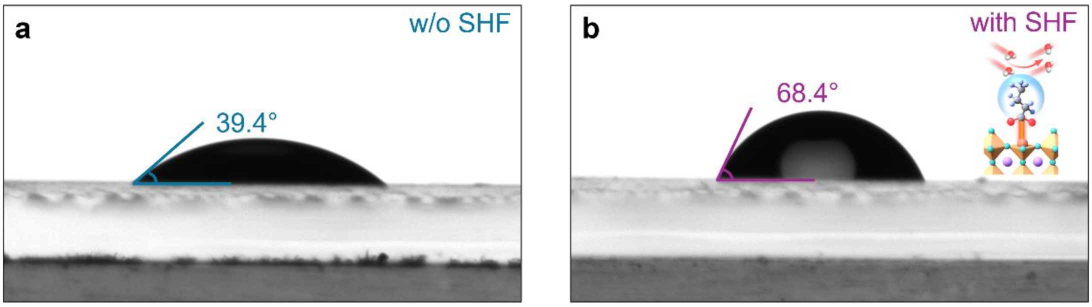

Figure S45. Water contact angles of the untreated (**a**) and SHF-treated (**b**) perovskite films.

The contact angle of water on the SHF-treated perovskite film is considerably higher than that of the control film, indicating the surface in the SHF-treated perovskite film is more hydrophobic and hence is resistant to moisture.[15]

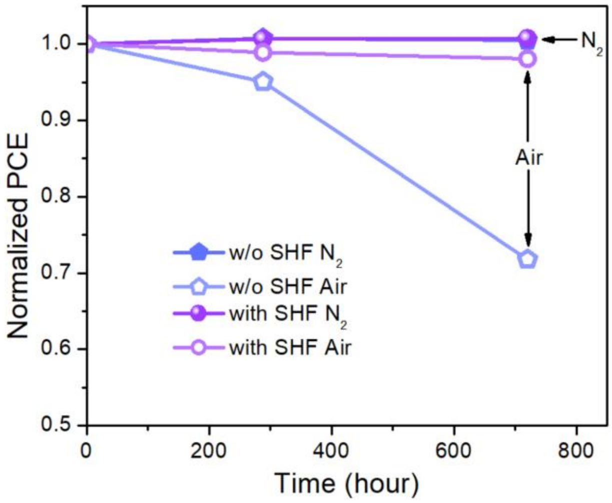

Figure S46. Humidity stability of unencapsulated control and SHF-treated devices (40% relative humidity).

In an N_2 atmosphere, both untreated and treated PSCs are stable after one month. However, when exposed to air with 40% relative humidity, a decrease in the efficiency of the control device was observed, retaining 72% of initial efficiency after 720 hours (**Figure S46**). The device treated with SHF maintained over 98% of the initial PCE over the same duration.

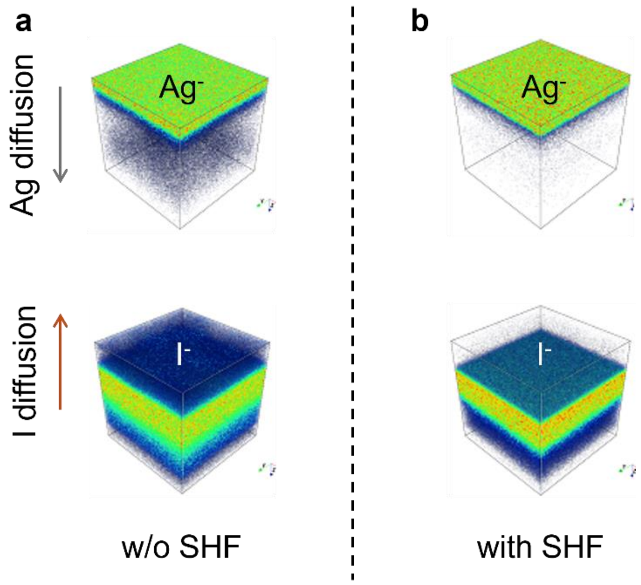

Figure S47. 3D Render of Ag and I ion diffusion for untreated (**a**) and SHF-treated (**b**) devices.

SHF effectively suppresses ion migration during device operation, protecting device stacks from degradation.

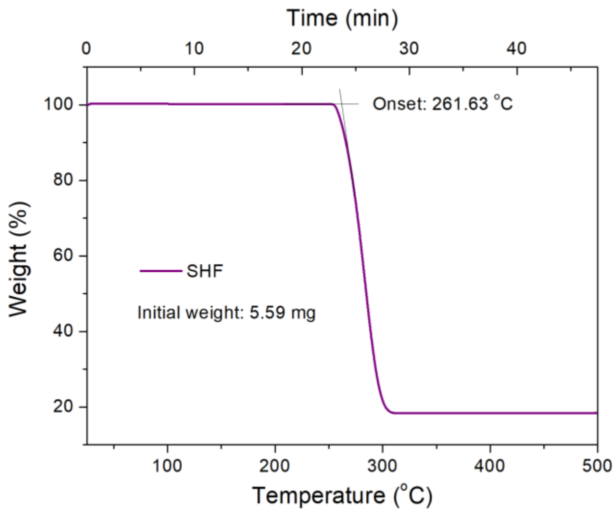

Figure S48. TGA curve of the SHF materials (heating rate 10 °C/min).

To examine the intrinsic thermal stability of SHF, we performed thermogravimetric analysis (TGA) to determine its decomposition temperature (**Figure S48**). The results show that SHF remains stable up to 260 °C, which is well above the temperatures applied during annealing and thermal stability tests in our experiments.

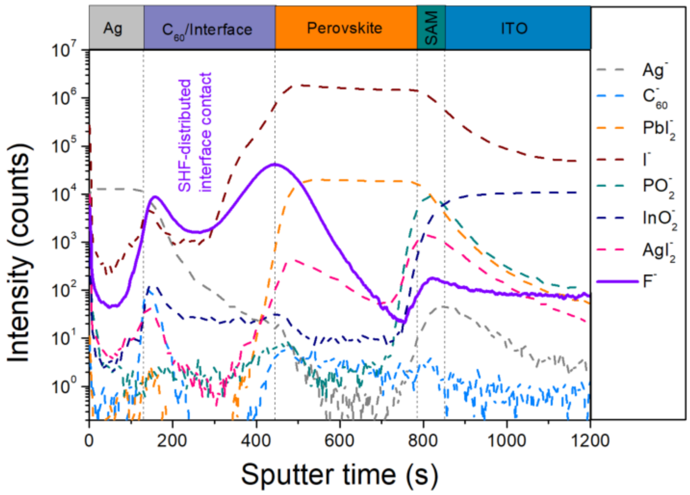

Figure S49. ToF-SIMS ion species depth profiles of Ag^- (Ag), C_{60}^- (C_{60}), PbI_2^- (perovskite), I^- (perovskite), PO_2^- (SAM), InO_2^- (ITO), AgI_2^- (AgI) and F^- (SHF) of the pristine SHF-treated device.

Supplementary tables

Table S1. Photovoltaic parameters extracted from the *J*-*V* curves of PSCs treated with different concentrations of SHF.

SHF concentration (mM)	V_{oc} (V)	J_{sc} (mA/cm^2)	FF (%)	PCE (%)
1	1.183	26.14	85.16	26.34
3	1.197	26.18	86.17	27.02
5	1.193	25.98	84.24	26.11

Table S2. Photovoltaic parameters extracted from the reverse and forward *J-V* curves of untreated and SHF-treated PSCs.

Devices	Sweeps	V_{oc} (V)	J_{sc} (mA/cm^2)	FF (%)	PCE (%)	HI (%)
untreated	Reverse	1.161	26.03	82.09	24.81	2.42
	Forward	1.154	25.96	80.83	24.21	
SHF-treated	Reverse	1.197	26.18	86.17	27.02	0.74
	Forward	1.197	26.14	85.72	26.82	

Table S3. Parameters used in the Driftfusion simulations.

The perovskite was assumed to be intrinsic and trap states (both bulk and interfacial) were assumed to lie midgap. Due to highly doped and selective nature of the ITO/SAM hole selective contact and to focus our simulations on the effects of SHF induced dipole shift, we have treated the perovskite/HTL interface as a Schottky diode (i.e., a perovskite metal interface where selectivity has been ensured by tuning the surface recombination velocities).

Parameter	Value
Perovskite Bandgap	1.55 eV
Perovskite Valance Band Energy	−5.4 eV
Perovskite Thickness	400 nm
Perovskite Carrier Mobility	10 $cm^2 V^{-1} s^{-1}$
Shockley-Read-Hall Lifetime	750 ns
Radiative Recombination Rate	1 x 10^{-11} $cm^{-3} s^{-1}$
Perovskite Relative Permittivity	25
Perovskite Effective Density of States	5 x 10^{18} cm^{-3}
Perovskite Mobile Ion Density	5 x 10^{16} cm^{-3}
ESC Bandgap	2.00 eV
ETL Conduction Band Energy (with SHF)	−4.15 (−3.95) eV
ETL Fermi Level (with SHF)	−5.15 (−4.95) eV
ETL Thickness	20 nm
ETL Carrier Mobility	10^{-3} $cm^2 V^{-1} s^{-1}$
ETL Relative Permittivity	3.5
ETL Effective Density of States	1 x 10^{20} cm^{-3}
Surface Recombination Velocity at the Perovskite/ETL Interface	0.5 cm s^{-1}
Surface Recombination Velocity of Electrons at the Perovskite/ETL Interface	0.5 cm s^{-1}
Cathode Work Function (with SHF)	−4.3 (−4.1) V
Surface Recombination Velocity of Electrons at the Perovskite/HTL Interface	0.1 cm s^{-1}
Surface Recombination Velocity of Holes at the Perovskite/HTL Interface	10^7 cm s^{-1}
Anode Work Function	−5.2 V

Supplementary References

1 Zizak, I. The mySpot beamline at BESSY II. *Journal of large-scale research facilities JLSRF* **2**, A102-A102 (2016).

2 Levine, I. *et al.* Charge transfer rates and electron trapping at buried interfaces of perovskite solar cells. *Joule* **5**, 2915-2933 (2021).

3 Milman, V. *et al.* Electronic structure, properties, and phase stability of inorganic crystals: A pseudopotential plane-wave study. *International Journal of Quantum Chemistry* **77**, 895-910 (2000). https://doi.org/https://doi.org/10.1002/(SICI)1097-461X(2000)77:5<895::AID-QUA10>3.0.CO;2-C

4 Perdew, J. P. *et al.* Restoring the Density-Gradient Expansion for Exchange in Solids and Surfaces. *Physical Review Letters* **100**, 136406 (2008). https://doi.org/10.1103/PhysRevLett.100.136406

5 Perdew, J. P., Burke, K. & Ernzerhof, M. Generalized Gradient Approximation Made Simple. *Physical Review Letters* **77**, 3865-3868 (1996). https://doi.org/10.1103/PhysRevLett.77.3865

6 Habisreutinger, S. N., Noel, N. K. & Snaith, H. J. Hysteresis Index: A Figure without Merit for Quantifying Hysteresis in Perovskite Solar Cells. *ACS Energy Letters* **3**, 2472-2476 (2018). https://doi.org/10.1021/acsenergylett.8b01627

7 Cao, Q. *et al.* Efficient and stable inverted perovskite solar cells with very high fill factors via incorporation of star-shaped polymer. *Science Advances* **7**, eabg0633 https://doi.org/10.1126/sciadv.abg0633

8 Cao, Q. *et al.* Environmental-Friendly Polymer for Efficient and Stable Inverted Perovskite Solar Cells with Mitigating Lead Leakage. *Advanced Functional Materials* **n/a**, 2201036 (2022). https://doi.org/https://doi.org/10.1002/adfm.202201036

9 Ho, K., Wei, M., Sargent, E. H. & Walker, G. C. Grain Transformation and Degradation Mechanism of Formamidinium and Cesium Lead Iodide Perovskite under Humidity and Light. *ACS Energy Letters* **6**, 934-940 (2021). https://doi.org/10.1021/acsenergylett.0c02247

10 Hu, J., Kerner, R. A., Pelczer, I., Rand, B. P. & Schwartz, J. Organoammonium-Ion-based Perovskites Can Degrade to Pb0 via Amine–Pb(II) Coordination. *ACS Energy Letters* **6**, 2262-2267 (2021). https://doi.org/10.1021/acsenergylett.1c00714

11 Wang, M. *et al.* Small Molecule Modulator at the Interface for Efficient Perovskite Solar Cells with High Short-Circuit Current Density and Hysteresis Free. *Advanced Electronic Materials* **6**, 2000604 (2020). https://doi.org/https://doi.org/10.1002/aelm.202000604

12 Wei, Z., Chen, H., Yan, K., Zheng, X. & Yang, S. Hysteresis-free multi-walled carbon nanotube-based perovskite solar cells with a high fill factor. *Journal of Materials Chemistry A* **3**, 24226-24231 (2015). https://doi.org/10.1039/C5TA07714A

13 Bati, A. S. R. *et al.* 1D-2D Synergistic MXene-Nanotubes Hybrids for Efficient Perovskite Solar Cells. *Small* **17**, 2101925 (2021). https://doi.org/https://doi.org/10.1002/smll.202101925

14 Yu, Z. *et al.* Intramolecular Electric Field Construction in Metal Phthalocyanine as Dopant-Free Hole Transporting Material for Stable Perovskite Solar Cells with >21 % Efficiency. *Angewandte Chemie International Edition* **60**, 6294-6299 (2021). https://doi.org/https://doi.org/10.1002/anie.202016087

15 Li, G. *et al.* Ionic Liquid Stabilizing High-Efficiency Tin Halide Perovskite Solar Cells. *Advanced Energy Materials* **11**, 2101539 (2021). https://doi.org/https://doi.org/10.1002/aenm.202101539
