# Supplementary material for: Stabilizing high-efficiency perovskite solar cells via strategic interfacial contact engineering
Source: Nat Photonics. 2025 Nov 7;20(1):55–62. doi: 10.1038/s41566-025-01791-1 (PMC12774855; doi:10.1038/s41566-025-01791-1)
Supplement: Supplementary file 2 — Reporting Summary [file 41566_2025_1791_MOESM2_ESM.pdf]

Corresponding author(s): Guixiang Li, Luyao Wang, Zhe Li, Meng Li, and Antonio Abate

Solar Cells Reporting Summary

Nature Portfolio wishes to improve the reproducibility of the work that we publish. This form is intended for publication with all accepted papers reporting the characterization of photovoltaic devices and provides structure for consistency and transparency in reporting. Some list items might not apply to an individual manuscript, but all fields must be completed for clarity.

For further information on Nature Research policies, including our data availability policy, see Authors & Referees.

Experimental design

Please check the following details are reported in the manuscript, and provide a brief description or explanation where applicable.

1. Dimensions

Area of the tested solar cells

☒ Yes ☐ No

The active areas of the tested solar cells are 0.0982 cm2 and 1 cm2, and aperture area for certification is 0.078 cm2.

Explain why this information is not reported/not relevant.

Method used to determine the device area

☒ Yes ☐ No

The device area is defined by metal aperture mask (black colour). Details in Methods Section.

Explain why this information is not reported/not relevant.

2. Current-voltage characterization

Current density-voltage (J-V) plots in both forward and backward direction

☒ Yes ☐ No

Provided in Figure S36.

Voltage scan conditions

☒ Yes ☐ No

The voltage conditions are forward (from -0.1 V to 1.3 V) and reverse (from 1.3 V to -0.1 V) scan with a speed of 20 mV/s and dwell time of 10 ms.

Explain why this information is not reported/not relevant.

Test environment

☒ Yes ☐ No

The J-V plots are tested in a N2 glovebox at room temperature with relative humidity below 20%.

Explain why this information is not reported/not relevant.

Protocol for preconditioning of the device before its characterization

☐ Yes ☒ No

Provide a description of the protocol.

No preconditioning was involved before test.

Stability of the J-V characteristic

☒ Yes ☐ No

We provided a MPP tracking, see Figures 4a.

Explain why this information is not reported/not relevant.

3. Hysteresis or any other unusual behaviour

Description of the unusual behaviour observed during the characterization

☐ Yes ☒ No

Provide a description of hysteresis or any other unusual behaviour observed during the characterization.

No unusual behaviour was observed.

Related experimental data

☐ Yes ☒ No

Provide a description of the related experimental data.

Not applicable.

4. Efficiency

External quantum efficiency (EQE) or incident photons to current efficiency (IPCE)

☒ Yes ☐ No

We provided EQE measurement in Supplementary Figure S37.

Explain why this information is not reported/not relevant.

A comparison between the integrated response under the standard reference spectrum and the response measure under the simulator

☒ Yes ☐ No

The difference between integrated JSC from EQE curves and JSC from J-V curves is within 1.3%, see Figure 3e and Supplementary Figure S37.

Explain why this information is not reported/not relevant.

For tandem solar cells, the bias illumination and bias voltage used for each subcell

- [ ] Yes — *Provide a description of the measurement conditions.*
- [x] No — No tandem solar cells wrer reported in our manuscript.

5. Calibration

Light source and reference cell or sensor used for the characterization

- [x] Yes — See details in Methods.
- [ ] No — *Explain why this information is not reported/not relevant.*

Confirmation that the reference cell was calibrated and certified

- [x] Yes — Our solar simulator was calibrated with a Silicon reference cell from the Fraunhofer-Institut für Solare Energiesysteme ISE.
- [ ] No — *Explain why this information is not reported/not relevant.*

Calculation of spectral mismatch between the reference cell and the devices under test

- [x] Yes — We calculated the mismatch factor, and we use the calculated value to correct the short-circuit current value in the J-V measurement.
- [ ] No — *Explain why this information is not reported/not relevant.*

6. Mask/aperture

Size of the mask/aperture used during testing

- [x] Yes — The sizes of aperture areas are 0.0982 cm2 and 1 cm2 for lab test and 0.078 cm2 for certification.
- [ ] No — *Explain why this information is not reported/not relevant.*

Variation of the measured short-circuit current density with the mask/aperture area

- [ ] Yes — *Report the difference in the short-circuit current density values measured with the mask and aperture area.*
- [x] No — No significant variations was observed.

7. Performance certification

Identity of the independent certification laboratory that confirmed the photovoltaic performance

- [x] Yes — The certificated PCE was obtained from an accredited Fujian Metrology Institute (National PV Industry Measurement and Testing Center).
- [ ] No — *Explain why this information is not reported/not relevant.*

A copy of any certificate(s)

- [x] Yes — A copy of certificate was provided in Supplementary Figures S39 and S40.
- [ ] No — *Explain why this information is not reported/not relevant.*

8. Statistics

Number of solar cells tested

- [x] Yes — Twenty solar cells per condition for statistics, see the caption of Figure 3d and Supplementary Figure S34.
- [ ] No — *Explain why this information is not reported/not relevant.*

Statistical analysis of the device performance

- [x] Yes — Provided in Figure 3d and Supplementary Figure S34.
- [ ] No — *Explain why this information is not reported/not relevant.*

9. Long-term stability analysis

Type of analysis, bias conditions and environmental conditions

- [x] Yes — The long-term operational stability through MPP tracking of the unencapsulated PSCs under continuous AM1.5G (100 mW/cm2) solar light in an N2 atmosphere at 20% RH and 25 °C (Figure 4a). The accelerated degradation measurements of unencapsulated PSCs were performed in an N2 atmosphere at 85 °C (Figure 4b). The thermal cycling tests were conducted between −40 °C and +85 °C (Figure 4c). An environmental stability of unencapsulated devices was conducted in a 40% relative humidity, see Supplementary Figure S46.
- [ ] No — *Explain why this information is not reported/not relevant.*
